# Arabidopsis exoribonuclease USB1 interacts with the PPR-domain protein SOAR1 to negatively regulate abscisic acid signaling

**DOI:** 10.1093/jxb/eraa315

**Published:** 2020-09-24

**Authors:** Yu Ma, Shang Zhang, Chao Bi, Chao Mei, Shang-Chuan Jiang, Xiao-Fang Wang, Zhi John Lu, Da-Peng Zhang

**Affiliations:** 1 MOE Key Lab of Bioinformatics, Center for Plant Biology, School of Life Sciences,Tsinghua University, Beijing, China; 6 University of Edinburgh, UK

**Keywords:** Abscisic acid signaling, *Arabidopsis thaliana*, early seedling growth, exoribonuclease USB1, pentatricopeptide repeat (PPR) protein SOAR1, spliceosome assembly

## Abstract

Signaling by the phytohormone abscisic acid (ABA) involves pre-mRNA splicing, a key process of post-transcriptional regulation of gene expression. However, the regulatory mechanism of alternative pre-mRNA splicing in ABA signaling remains largely unknown. We previously identified a pentatricopeptide repeat protein SOAR1 (suppressor of the ABAR-overexpressor 1) as a crucial player downstream of ABAR (putative ABA receptor) in ABA signaling. In this study, we identified a SOAR1 interaction partner USB1, which is an exoribonuclease catalyzing U6 production for spliceosome assembly. We reveal that together USB1 and SOAR1 negatively regulate ABA signaling in early seedling development. USB1 and SOAR1 are both required for the splicing of transcripts of numerous genes, including those involved in ABA signaling pathways, suggesting that USB1 and SOAR1 collaborate to regulate ABA signaling by affecting spliceosome assembly. These findings provide important new insights into the mechanistic control of alternative pre-mRNA splicing in the regulation of ABA-mediated plant responses to environmental cues.

## Teaser

USB1, an exoribonuclease catalyzing U6 production for spliceosome assembly, negatively regulates, cooperatively with SOAR1, ABA signaling in early seedling development, probably by affecting spliceosome assembly.

## Introduction

Post-transcriptional regulation plays crucial roles in the life cycle of eukaryotes (Halbeisen*et al.*, 2008). As a key process of this regulation, pre-mRNA splicing involves excising introns and joining together exons to form the mature mRNA. This process is catalyzed by the spliceosome, a large molecular complex consisting of five small nuclear ribonucleoprotein particles (snRNPs) and a multitude of non-snRNP proteins in human and yeast. The snRNPs are composed of small nuclear uridine-rich RNAs (U1, U2, U4, U5, and U6 snRNAs) and their corresponding interacting proteins (Jurica and Moore, 2003; Will and Luhrmann, 2011; Fica and Nagai, 2017). The splicing reaction is initiated by the recruitment of U1snRNP to the 5' splice site (5' SS) of pre-mRNA. Splicing factor 1 and the 35 kDa U2 auxiliary factor subunit bind to the branch point (BP) sequence and the polypyrimidine tract of the intron, respectively, which aids U2 snRNP binding to form the pre-spliceosome (A complex), and further recruits the U4/U6·U5 tri-snRNP to generate the pre-catalytic complex B. The B complex is assembled into the B^act^ complex, which further reorganizes into the B* complex. The B* complex catalyzes the first of the transesterification reactions. This process generates complex C, which causes a cleaved 5' SS and lariat intron. Further, the conversion of the complex to C* promotes the second catalytic step, resulting in cleavage of the 3' splice site (3' SS) and formation of a post-spliceosomal complex. Finally, exons are joined together to form the mature mRNA, introns are released, and snRNPs are recycled (Will and Luhrmann, 2011; Fica and Nagai, 2017; Shi, 2017). It is particularly noteworthy that the intramolecular stem–loop of U6 snRNA, the helix I of the U2/U6 duplex, loop I of U5 snRNA, and the catalytic magnesium ions are components of the core active site of the spliceosome (Shi, 2017).

In plants, the process by which the spliceosome is assembled is still unclear. Recent studies, however, revealed various regulatory mechanisms of alternative splicing. Chromatin structure, RNA polymerase II elongation rate, serine/arginine-rich proteins, and heterogeneous nuclear RNPs (hn RNPs) may be involved in the recognition of splicing sites and spliceosome activity (Luco *et al.*, 2011; Koncz *et al.*, 2012;Brzyzek and Swiezewski, 2015; Nieto Moreno *et al.*, 2015; Romero-Barrios *et al.*, 2018). Differences may exist in splicing machineries and sequence recognition between plants and animals. The average size of introns in animals (~5 kb) is longer than that in plants (~160 bp), but the consensus sequences of the 5' SS, 3' SS, and BP are similar in animals and plants (Simpson *et al.*, 2002; Sakharkar *et al.*, 2004; Iwata and Gotoh, 2011). Alternative splicing, which generally includes five categories of events, namely intron retention, exon skipping, 5' SS alternative splicing, 3' SS alternative splicing, and mutually exclusive exon splicing, generates multiple mature mRNA isoforms from the same pre-mRNA. There are ~95% of intron-containing genes in humans and >61% of intron-containing genes in *Arabidopsis thaliana* that are alternatively spliced, whereas the most frequent alternative splicing event in human is exon skipping but in plants is intron retention (~40%) (Pan *et al*., 2008; Marquez *et al.*, 2012; Reddy *et al.*, 2013).

The phytohormone abscisic acid (ABA) regulates multiple aspects of plant development and plant adaption to environmental stress (reviewed in Finkelstein *et al.*, 2002; Adie *et al.*, 2007; and Cutler *et al.*, 2010). There is mounting evidence that alternative splicing plays a crucial role in ABA signaling (reviewed in Laloum *et al.*, 2018). HAB1 is a member of the clade-A type-2C protein phosphatases (PP2Cs), which negatively regulates ABA signaling (Umezawa *et al.*, 2009; Vlad *et al.*, 2009). The *HAB1* pre-mRNA undergoes alternative splicing to produce two functional splice variants *HAB1.1* and *HAB1.2* that function oppositely to regulate ABA signaling (Z. Wang *et al.*, 2015; Zhan *et al.*, 2015). The ROA1/RBM25 (Regulator of ABA Response), a homolog of human splicing factor RBM25, regulates alternative splicing of *HAB1*and numerous other genes that participate in ABA signaling (Z. Wang *et al.*, 2015; Zhan *et al.*, 2015). The two mRNA cap-binding proteins CBP20 and CBP80 function as negative regulators of ABA responses by influencing alternative splicing of the first intron in most genes, particularly at the 5' SS in Arabidopsis (Hugouvieux *et al.*, 2001; Papp *et al.*, 2004; Raczynska *et al.*, 2010). Additionally, two ABA-responsive genes, *ABI5* and *CIPK3*, also undergo alternative pre-RNA splicing by which these two genes function to affect ABA signaling (Zou *et al*., 2007; Sanyal *et al.*, 2017*a*; Xiong *et al.*, 2019).

USB1 (U6 biogenesis protein 1) is a 3'–5' exoribonuclease belonging to the 2H phosphodiesterase superfamily that shortens the poly(U) tail of U6 snRNA in most eukaryotes including human, yeast, and maize (Mroczek *et al.*, 2012; Shchepachev *et al.*, 2012; Hilcenko *et al.*, 2013; Didychuk *et al.*, 2017; Li *et al.*, 2017). In humans, processing of U6 by USB1 creates a terminal 2', 3'-cyclic phosphate which stimulates binding of U6 to a subunit of the spliceosome, the Sm-like (LSm) proteins 2–8, to form the U4/U6 snRNPs (Licht *et al.*, 2008; Hilcenko *et al.*, 2013; Didychuk *et al.*, 2016). The Arabidopsis homologues of human LSm, namely LSM2–LSM8, form a complex where LSM8 is a component of the U6 snRNP and were shown to be required for precursor mRNA splicing through U6 snRNA stabilization, which plays an important role in the environment-dependent regulation of spliceosome activity (Perea-Resa *et al.*, 2012; Carrasco-Lopez *et al*., 2017; Huertas *et al.*, 2019). The LSM1–LSM7 complex was reported to differentially regulate Arabidopsis tolerance to abiotic stress conditions by promoting selective mRNA decapping and especially by targeting to NCED3 and NCED5, two key enzymes in ABA biosynthesis (Perea-Resa *et al.*, 2016). The Arabidopsis LSM4 and LSM5 were reported to function in ABA and salt stress responses by affecting alternative splicing (Xiong *et al.*, 2001; Zhang *et al.*, 2011; Cui *et al.*, 2014). In maize, a loss-of-function mutation in the *USB1* gene results in abnormal 3' ends of U6 snRNAs and splicing defects of many genes, affecting seed development (Li *et al.*, 2017). However, the regulatory mechanism of alternative pre-mRNA splicing by which plants regulate ABA signaling remains largely unknown.

We previously identified the pentatricopeptide repeat (PPR) protein SOAR1 (for Suppressor of the ABAR-overexpressor 1) as a crucial player (Mei *et al.*, 2014; Jiang *et al*., 2014, 2015) downstream of the ABAR (the putative ABA receptor; Shen *et al.*, 2006; Wu *et al.*, 2009) in ABA signaling. In this study, we identified a SOAR1interaction partner, USB1, which is an exoribonuclease catalyzing U6 production, and revealed that USB1 cooperates with SOAR1 to negatively regulate ABA signaling in early seedling development, probably by affecting spliceosome assembly. USB1 and SOAR1 are both required for the splicing of transcripts of many genes, including those involved in ABA signaling and salt response pathways. These findings reveal the mechanistic basis for alternative pre-mRNA splicing in the regulation of ABA-mediated plant responses to environmental cues.

## Materials and methods

### Plant materials and growth conditions

The Arabidopsis Columbia-0 (Col-0) ecotype was used as the wild type and all mutant background materials. The T-DNA insertion line of the *usb1-1* (SAIL_717_G03) mutant in the *USB1* gene (Arabidopsis genomic locus At5g51170) was obtained from the Arabidopsis Biological Resource Center (ABRC). The *usb1-2* mutant was generated using CRISPR/Cas9 [clustered regularly interspaced palindromic repeats (CRISPR)/CRISPR-associated protein 9] technology as described (Z.P. Wang *et al.*, 2015). A single guide RNA target C1 in the *USB1* gene was cloned into the pHEE2A-TRI vector. The resulting plasmid was introduced into *Agrobacterium tumefaciens* strain GV3101 and transformed by floral infiltration into Arabidopsis wild-type (Col-0) plants. T_1_ transgenic plants were screened by hygromycin resistance and confirmed by DNA sequencing. The homozygous T_3_ generation seeds were selected for further analysis. The seeds of the *soar1-2* mutant were obtained from the Versailles Genetics and Plant Breeding Laboratory, *Arabidopsis thaliana* Resource Centre (INRA) and identified as a knockdown allele as described previously (Mei *et al.*, 2014; Jiang *et al.*, 2015). The primers for identification of these mutants are listed in Supplementary Table S1 at *JXB* online.

To generate transgenic lines overexpressing the *USB1* gene, the ORF sequence of the *USB1* gene was amplified by PCR and cloned into the binary vector PMDC85 with a green fluorescent protein (GFP) tag under control of the *Cauliflower mosaic virus* (CaMV) 35S promoter. The resulting plasmid (*USB1*-PMDC85) was introduced into *A. tumefaciens* strain GV3101 and transformed by floral infiltration into Arabidopsis wild-type (Col-0) plants. Transgenic plants with a single T-DNA insertion were screened by hygromycin resistance and confirmed by quantitative real-time PCR (qRT-PCR). T_3_ generation seeds of two homozygous overexpression lines, OE1 and OE3, were selected for further analysis. The above plasmid (*USB1*-PMDC85) was also transformed into *usb1-1* and *usb1-2* mutants to generate complementation lines. All of the primers used for generation of the transgenic plants are presented in Supplementary Table S1.

The *usb1-1 soar1-2* and *usb1-2 soar1-2* double mutants were generated by genetic crossing and identified by PCR genotyping. For the generation of the *USB1*overexpression plant in the *soar1-2* mutant background, the overexpressed *USB1* gene was introduced into *soar1-2* by crossing the USB1-OE3 line and *soar1-2* plants. For the generation of the *SOAR1* overexpression plant in the *usb1* mutant background, the previously generated *SOAR1* overexpression line OE6 (expressing SOAR1 fused with GFP; Mei *et al.*, 2014; Jiang *et al.*, 2015) was used, and this overexpressed *SOAR1* in SOAR1-OE6 was introduced to *usb1-1* and *usb1-2* by crossing. Plants were grown in a growth chamber at 22 °C on Murashige and Skoog (MS) medium (MS salt, 2% sucrose, 0.8% agar, pH 5.7–6.0) in a growth chamber at ~80 μmol photons m^−2^ s^−1^ or in compost soil at~120 μmol photons m^−2^ s^−1^lit by cool-white fluorescent lamps under a 16 h light/8 h dark photoperiod and 60% relative humidity.

### Phenotypic analysis

The phenotypic analysis was performed essentially as described previously (Shang *et al.*, 2010). For the seedling growth assay, two approaches were used to test the effects of ABA, NaCl, or mannitol on early seedling growth. The first approach is that seeds were sown on basal MS medium or MS medium supplemented with different concentrations of (±)-ABA (Sigma, A1049, Saint Louis, MO, USA), NaCl (Amresco, 0241, OH, USA), or d-mannitol (Amresco, 0122, OH, USA) medium. After 3 d of stratification at 4 °C, they were placed in a light incubator for 10 d, and the root length was recorded. The second approach is that seeds were planted in ABA-free MS medium and, after 3 d of stratification at 4 °C, they were placed in a light incubator for 60 h, and then young seedlings were transferred to (±)-ABA-containing MS medium and continued to grow for 10 d before the root length was recorded. For the cotyledon greening assay, seeds were sown on either (±)-ABA-free MS medium or MS medium supplemented with different concentrations of (±)-ABA for 3 d of stratification at 4 °C, and then the germinating seeds/young seedlings were placed in a light incubator for 10 d before the cotyledon greening rates were recorded. Cotyledon greening was defined as obvious cotyledon expansion and turning green.

### Luciferase complementation imaging assay

The luciferase (Luc) complementation imaging (LCI) assays were performed essentially as previously described (Shang *et al.*, 2010). The full-length coding sequences of the *USB1* and *SOAR1* gene were cloned, respectively, in the N-terminal half (NLuc) or C-terminal half (CLuc) of the LCI vector (USB1–NLuc and CLuc–USB1, or SOAR1–NLuc and CLuc–SOAR1). The resulting plasmids were transferred into *A. tumefaciens* strain GV3101, and infiltrated into young but fully expanded leaves of 7-week-old tobacco (*Nicotiana benthamiana*) leaves using a needleless syringe. After infiltration, plants were grown in the dark for 24 h and then under a 16 h light/8 h dark photoperiod for 48–60 h. The Luc activity was observed with a CCD imaging apparatus (AndoriXon, UK). The primers used for constructing the related plasmids are listed in Supplementary Table S1.

### Bimolecular fluorescent complementation assay in Arabidopsis protoplasts and tobacco leaves

The bimolecular fluorescent complementation (BiFC) assays were performed essentially as previously described (Shang *et al.*, 2010). Yellow fluorescent protein (YFP) was used for the BiFC assays. The coding region of the *USB1* or *SAOR1* gene was cloned into the pUC-SPYNE vector (Walter *et al.*, 2004) harboring the N-terminal half of YFP (NYFP) or the C-terminal half of YFP (CYFP) to form USB1–NYFP and USB1–CYFP, or SOAR1–NYFP and SOAR1–CYFP. The resulting plasmids were co-transformed into Arabidopsis wild-type (Col-0) mesophyll protoplasts by the polyethylene glycol-mediated transient transformation protocol (Yoo *et al.*, 2007). The YFP fluorescence was imaged under a confocal laser scanning microscope (Zeiss LSM780, Germany).

To study the subcellular localization of the interaction between SOAR1 and USB1, we performed the BiFC assay in tobacco (*N. benthamiana*) leaves as described previously (Jia *et al.*, 2016). The coding regions of *USB1* and *SOAR1* were fused to the NYFP and CYFP, respectively, to form USB1–NYFP and SOAR1–CYFP. The resulting plasmids were transferred into *A. tumefaciens* strain GV3101, and infiltrated into young but fully expanded leaves of 7-week-old tobacco leaves using a needleless syringe. After infiltration, plants were grown in the dark for 24 h and then under a 16 h light/8 h dark photoperiod for 48–60 h. The YFP fluorescence was imaged under a confocal laser scanning microscope (Zeiss LSM780, Germany). The primers used for constructing the related plasmids are listed in Supplementary Table S1.

### Co-IP assay

The Co-IP assay was performed essentially as previously described (Shang *et al.*, 2010). Total proteins of transgenic plants (USB1-OE3) expressing USB1–GFP fusion protein were extracted, with the extraction buffer consisting of 50 mM Tris–HCl (pH 7.4), 150 mM NaCl, 1 mM EDTA, 1%(v/v) TritonX-100, 10% (v/v) glycerol, 1× protease inhibitor cocktail (Roche, 4 693 159 001, Germany), 1 mM phenylmethylsulfonyl fluoride (PMSF), and 1mM DTT. The total proteins were incubated with the anti-GFP mAb-Magnetic Beads (MBL, D153-11, Japan) with rotating at 4°C for 12 h. The beads were collected and washed six times with the pre-cooled extraction buffer and then re-suspended with 2 vols of the protein loading buffer containing 100 mM Tris–HCl (pH 6.8), 4% (w/v) SDS, 0.1% (w/v) bromophenol blue, 20% (v/v) glycerol, and 5% (v/v) β-mercaptoethanol. The immunoprecipitates and the input were separated on a 10% SDS-PAGE gel, immunoblotted with the anti-SOAR1 or anti-GFP antibodies, and detected using Clarity™ Western ECL Substrate (Bio-Rad, 1 705 060, USA). The primers used for the vector constructions are shown in Supplementary Table S1.

### *In vitro* pull-down assay

The full-length coding regions of *SOAR1* (linked to *His*) and *USB1* (linked to *GST*) were amplified by PCR and cloned into the pMAL-c5X vector (NEB, N8108S, USA) and pGEX4-T-1 (Pharmacia) vector, respectively. The constructions were transformed into *Escherichia coli* BL21 (DB3) to produce SOAR1-His, USB1–glutathione *S*-transferase (GST), or GST protein. The GST and USB1–GST proteins were purified from *E*. *coli* using glutathione beads 4FF (Smart-life sciences, SA010010, China) according to the manufacturer’s instructions, and the SOAR1-His proteins were purified using Ni Sepharose 6 Fast Flow agarose (GE, 17-5318-06, USA). The USB1–GST and GST were incubated with glutathione–magarose beads (Smart-life sciences, M00201, China) rotating at 4°C for 2 h in incubation buffer consisting of 50 mM Tris–HCl (pH 7.5), 50 mM NaCl, 10% (v/v) glycerol, 0.1% (v/v) Tween-20, 0.15% (v/v) β-mercaptoethanol, 25 mM imidazole, and 1× protease inhibitor cocktail. The beads were collected and washed three times with incubation buffer on ice. Then, the SOAR1-His fusion protein was incubated with the above-washed beads in the incubation buffer rotating at 4°C for another 2 h. The beads were collected and washed six times with the incubation buffer and re-suspended in 2 vols of the protein loading buffer. The immunoprecipitates and input were separated on a 10% SDS–PAGE gel and immunoblotted with anti-GST or anti-His antibodies. The primers used for constructing the related plasmids are listed in Supplementary Table S1.

### Subcellular localization of the USB1 protein

The subcellular localization analysis was performed essentially as described previously (Mei *et al.*, 2014). The coding region of the *USB1* linked to *GFP* was cloned into the pROK219 vector, driven by the CaMV 35S promoter (*35S::USB1-GFP*). SOAR1 was used as a marker for cytosolic–nuclear localization (Mei *et al.*, 2014) and the bHLH (basic helix–loop–helix) transcription factor FBI1/HFR1 was used as a nuclear-localized marker (Fairchild *et al.*, 2000; Jang *et al.*, 2005). The *35S::USB1-GFP* plus *SOAR1-RFP*(red fluorescence protein) or *35S::USB1-GFP* plus *FBI1-RFP* were transiently co-expressed in Arabidopsis protoplasts using the polyethylene glycol-mediated transformation protocol (Yoo *et al.*, 2007). The fluorescence signal of GFP and RFP was detected using a confocal microscope (Zeiss LSM780, Germany). The *35S::USB1-GFP* plasmid was also transformed into onion epidermal cells by particle bombardment-mediated transformation with a gene gun system (Bio-Rad). The samples were cultured at 26 °C for 16 h, and then observed with a confocal laser scanning microscope (Zeiss, LSM780, Germany). In addition, the subcellular localization of USB1 was also assayed in the complementation lines (Com11-1) expressing *35S::USB1-GFP* in the *usb1-1* background. The fluorescence signals of GFP and DAPI (for nuclear staining) were imaged under a confocal laser scanning microscope (Zeiss LSM780, Germany). The primers for construction are listed in Supplementary Table S1.

### qRT-PCR

Total RNA was extracted from 2-week-old plants with a Total RNA Rapid Extraction Kit (BioTeke, RP1202, China), treated with RNase-free DNase I (NEB, M0303S, USA) to degrade genomic DNA, and then purified using an RNA Purification Kit (BioTeke, RP1802, China) according to the manufacturer’s instructions. A 5 μg aliquot of RNA was used for first-strand cDNA synthesis using the Transcriptor cDNA synthesis Kit (Roche, 4 897 030 001, Germany). The primers of a series of ABA-responsive genes used for qRT-PCR are listed in Supplementary Table S1. Analysis was performed using the Real-Time System CFX96™ C1000 Thermal Cycler (Bio-Rad, USA). All experiments were repeated at least three times along with three independent repetitions of the biological experiments. *ACTIN2/8* genes were used as internal controls.

### RNA sequencing (RNA-seq)

For the RNA-seq experiments, total RNA was extracted from 10-day-old plants, treated with RNase-free DNase I, and purified as described above. mRNA-seq libraries were constructed by following the standard Illumina protocol with three biological replicates per genotype. The Illumina sequencing was performed with an Illumina HiSeq 2500 paired-end system. For each sample, RNA-seq raw reads were trimmed using cutadapt v1.16 (Martin *et al.*, 2011) to remove the potential Illumina adaptor contamination and conduct read trimming and clipping of the low quality bases. The remaining reads were aligned to the *A. thaliana* genome sequence and the reference-annotated genes (TAIR10) using STAR v2.5.3a (Dobin *et al.*, 2013). Based on the RNA-seq mapped reads and the reference-annotated transcripts, featureCountsv1.6.2 (Liao *et al.*, 2014) was used to calculate the gene counts. rMATS (Shen *et al.*, 2014) was used to detect differential alternative splicing events for our RNA-seq samples. Among the alternative spliced events, the absolute value of the Inc Level Difference>0.05 and *P*-value<0.05 showed significantly and differentially alternative splice events. Rmats2sashimiplot was used to convert the alternative splicing analysis output into splicing visualization.

### Measurement of intron retention and splicing efficiency

For assaying the intron retention and splicing efficiency, total RNA was extracted from 10-day-old plants, treated with RNase-free DNase I, and then purified as described above. A 5μg aliquot of RNA was used for first-strand cDNA synthesis with the Transcriptor cDNA synthesis Kit (Roche, 4 897 030 001, Germany). The relative unspliced mRNA level was tested by qRT-PCR using unspliced primers. The relative spliced mRNA level was tested by qRT-PCR using spliced primers. The splicing efficiency was determined by calculating the ratios of the level of spliced mRNA relative to the level of total mRNA (spliced plus unspliced mRNA). *ACTIN2/8* genes were used as internal controls. The primers to amplify spliced and unspliced mRNA are listed in Supplementary Table S1. All the experiments were repeated at least three times along with three independent repetitions of the biological experiments.

### Gene ontology analysis

The gene ontology (GO) analysis was conducted using the agriGO platform, which is a GO analysis toolkit specifically focused on agricultural species. The singular enrichment analysis (SEA) was chosen as the analysis tool and the suggested *A. thaliana* backgrounds were selected as the reference. The hypergeometric statistical test and Bonferroni multitest adjustment were used as the statistical test methods. The significance level threshold is 0.05; five was set as the minimum number of mapping entries (Tian *et al.*, 2017).

## Results

### USB1 interacts with SOAR1 in the nucleus and cytoplasm

**Fig. 1.**
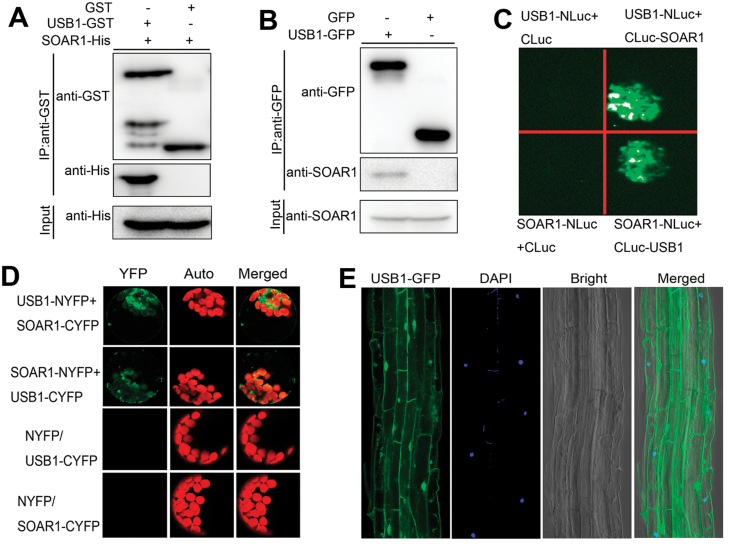
USB1 co-localizes and physically interacts with SOAR1 in both the nucleus and cytoplasm. (A) *In vitro* pull-down assay to test the direct interaction between SOAR1 and USB1 protein. The SOAR1-His proteins were incubated with immobilized GST or USB1–GST proteins, and the antibody against GST tag (IP:anti-GST) was used to pull down the purified USB1–GST or GST protein. Both the immunoprecipitated fractions and input were tested by immunoblotting with anti-His and anti-GST antibodies, respectively. (B) Co-immunoprecipitation (Co-IP) assay to test the interaction of USB1 with SOAR1 in Arabidopsis. Total proteins, extracted from the homozygous transgenic plants expressing GFP or USB1–GFP fusion protein (USB1–OE3), were immunoprecipitated with anti-GFP antibody (IP:anti-GFP). The immunoprecipitates and the input were tested by immunoblotting with anti-SOAR1 and anti-GFP antibodies. (C) Luciferase complementation imaging (LCI) assay to test the interaction between USB1 and SOAR1 using the *N. benthamiana* system. The coding sequences of *USB1*or *SOAR1* were cloned into the N-terminal fragment of Luc (NLuc) and the C-terminal fragment of Luc (CLuc) to form USB1–NLuc(or SOAR1–NLuc) and CLuc–USB1 (or CLuc–SOAR1), respectively. The constructs pairs of USB1–NLuc/CLuc–SOAR1 and CLuc–USB1/SOAR1–NLuc were co-injected into *N. benthamiana* leaves, and the Luc signals were observed 72 h after infiltration. The combinations of USB1–NLuc/CLuc and SOAR1–NLuc/CLuc vectors were used as negative controls. (D) Bimolecular fluorescent complementation imaging (BiFC) assay using yellow fluorescent protein (YFP) to test the interaction between USB1 and SOAR1 in Arabidopsis mesophyll protoplasts. The coding region of *USB1* or *SOAR1* was fused to the N-terminus of YFP (USB1–NYFP or SOAR1–NYFP), or to the C-terminus of YFP (USB1–CYFP or SOAR1–CYFP). The constructs pairs (as indicated) were co-transformed into Arabidopsis wild-type (Col-0) mesophyll protoplasts. The combinations of NYFP /SOAR1–CYFP or USB1–CYFP were used as negative controls. The YFP fluorescence was imaged under a confocal laser scanning microscope. YFP, YFP signal; Auto, chloroplast autofluorescent signal; Merged, merged image of the YFP signal with the chloroplast autofluorescent signal. (E) Transgenic expression of the USB1–GFP fusion protein in whole Arabidopsis plants, showing that the USB1–GFP fusion protein (left) is localized to both the nucleus and cytoplasm in the root of a transgenic complementation line (the line Com11-1, see Supplementary Fig. S5) expressing 35S::*USB1-GFP* in the *usb1-1* background. Note that the expression level of *USB1* in the Com11-1 transgenic line is slightly higher than that in the wild-type plants (Supplementary Fig. S5). The nuclei are indicated by DAPI staining. Bright, bright field; Merged, merged image of the DAPI signal with the USB1–GFP signal in the bright field. (F) Transient expression of the USB1–GFP fusion protein in the Arabidopsis protoplasts, showing the nuclear–cytoplasmic dual localization of USB1. Left panels: the signal of the USB1–GFP fusion protein overlaps the signal of a nuclear-localized bHLH (basic helix–loop-helix) transcription factor FBI1/HFR1 (FBI1–RFP) tagged with mCherry (a red fluorescent protein) in the nuclear portion (Merged, merged image of USB1–GFP with FBI1–RFP in the bright field). Right panels: the signal of the USB1–GFP fusion protein completely overlaps the signal of the cytosolic–nuclear dual-localized SOAR1–RFP (SOAR1 tagged with mCherry) fusion protein (Merged, merged image of USB1–GFP with SOAR1–RFP in the bright field). Bright, bright-field. (G) BiFC assays in the *N. benthamiana* leaves, indicating that USB1 interacts with SOAR1 in both the nucleus and cytoplasm. The coding regions of *USB1* and SOAR1 were fused to the N-terminus of YFP and the C-terminus of YFP to form USB1–NYFP and SOAR1–CYFP, respectively. The construct pairs (as indicated) were co-infiltrated into *N. benthamiana* leaves. YFP fluorescence was detected in *N. benthamiana* leaves co-infiltrated with combinations of USB1–NYFP and SOAR1–CYFP. The combination of NYFP/SOAR1–CYFP was used as a negative control. The YFP fluorescence was imaged under a confocal laser scanning microscope. YFP, YFP signal; DAPI, staining for the nucleus; Merged, merged image of the YFP signal with the DAPI signal. All the experiments were repeated five times with similar results.

We previously showed that the PPR-domain protein SOAR1 plays a crucial role in ABA signaling (Mei *et al.*, 2014; Jiang *et al*., 2014, 2015; Bi *et al.*, 2019). In a yeast two-hybrid screen for SOAR1-interacting proteins, we found that Arabidopsis USB1, a homolog of USB1in yeast and human (Supplementary Fig. S1), is a potential SOAR1-interaction partner. We confirmed the interaction between USB1 and SOAR1 with four independent experiments. In an *in vitro* pull-down assay, the SOAR1-His fusion protein was pulled down by anUSB1–GST fusion protein but not by the GST tag alone (a negative control) (Fig. 1A). In an *in planta* Co-IP assay with the total proteins extracted from two transgenic plants expressing GFP or USB1–GFP fusion protein, SOAR1 protein was co-immunoprecipitated by USB1–GFP but not by GFP (a negative control) (Fig. 1B). In the tobacco LCI assay, co-expression of SOAR1 plus USB1 in tobacco (*N. benthamiana*) leaves resulted in strong Luc fluorescence, whereas no Luc activity was detected in the negative controls (Fig. 1C). In the BiFC assay, the fluorescence signal appeared when SOAR1 plus USB1 were co-expressed in Arabidopsis protoplasts, but no signal was found with the negative controls (Fig. 1D). All these data consistently indicate that USB1 specifically interacts with SOAR1.

**Fig. 2.**
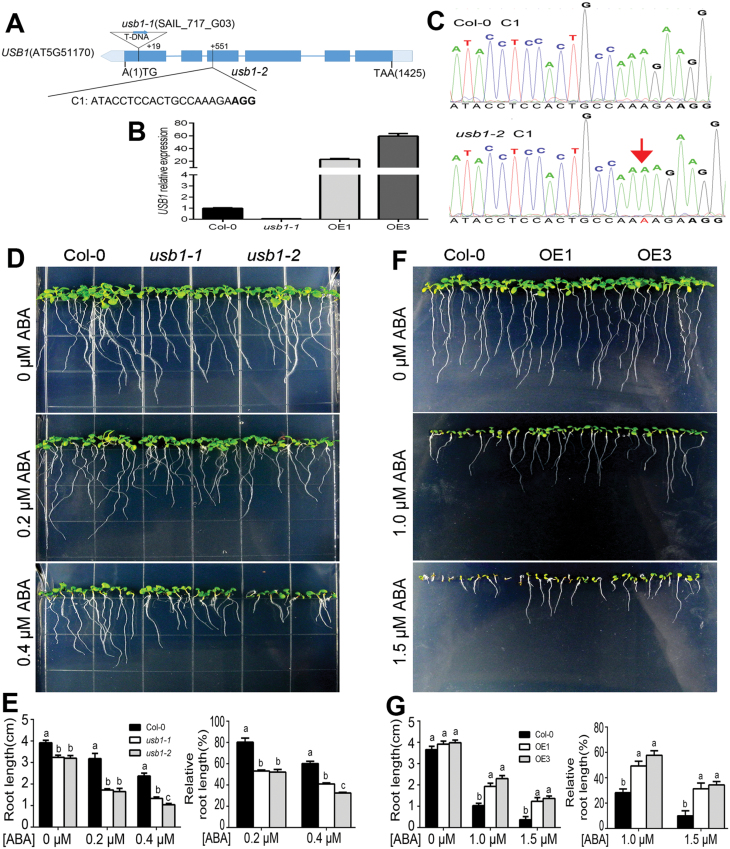
USB1 negatively regulates ABA signaling in early seedling growth. (A–C) Identification of the *usb1* mutants. (A) For the *usb1-1* mutant (with Col-0 background; SAIL_717_G03 from ABRC), the T-DNA was inserted into the first exon of the At5G51170 genome locus at 19 bp downstream of the start codon (ATG). Boxes and lines represent exons and introns, respectively. The *usb1-2* mutant was generated by CRISPR/Cas9 technology. The target site (C1) for CRISPR/Cas9 technology is shown. Protospacer adjacent motifs (PAMs) are marked with bold letters. (B) qRT-PCR analysis of the *USB1* expression level in seedlings of the 2-week-old wild-type Col-0, *usb1-1*, and *USB1* overexpression lines OE1 and OE3. *ACTIN2/8* genes were used as internal controls. The expression level of *USB1* in Col-0 was standardized as one unit. Each value is the mean ±SE of three independent biological determinations. (C) The mutation in *usb1-2*, which was evaluated by sequencing. An adenosine insertion of the *usb1-2* mutant in the *USB1* gene locus at 551 bp downstream of ATG is indicated by a red letter A and an arrow. (D–G) Early seedling growth of wild-type Col-0, *usb1-1*and *usb1-2* mutants (D, E), and Col-0, OE1 and OE3 *USB1* overexpression lines (F, G). Seeds were directly planted in ABA-free MS medium (0 μM) or MS medium supplemented with different concentrations of (±)-ABA (0.2 μM and 0.4 μM in D and E, and 1.0μM and 1.5 μM in F and G), and the seedling growth was investigated 10 d after stratification at 4 °C for 3 d. (E, G) Statistical analysis of root length (left) and relative root length (right) of different genotypes described in (D) and (F), respectively. Relative values of the root length of each genotype grown on MS medium containing 0.2μM and 0.4 μM (D, E) or 1.0 μM and 1.5 μM (F, G) (±)-ABA were normalized relative to the value of the corresponding genotype at 0 μΜ ABA, which was taken as 100%. In (E, G), each value is the mean ±SE of five biological determinations, and different letters represent significant differences at *P*<0.05 (Duncan’s multiple range test) when comparing values within the same ABA concentration.

We conducted transgenic expression of the USB1–GFP fusion protein in Arabidopsis T-DNA insertion knockout mutant plants (*usb1-1*; Fig. 2A, B), and selected a transgenic line (Com11-1; Supplementary Fig. S5) in which the expression level of *USB1* is slightly higher than that of the wild-type plants to investigate USB1 subcellular localization. We observed that the USB1 protein is localized to both the nucleus and cytoplasm in root cells (Fig. 1E). The transient expression of the USB1–GFP fusion protein in both Arabidopsis protoplasts and onion epidermal cells verified the nuclear–cytoplasmic dual localization of USB1(Fig. 1F; Supplementary Fig. S2A), and showed that USB1 co-localizes to the nucleus and cytoplasm with SOAR1 (Fig. 1F). We also showed that the *USB1* gene is expressed in nearly all tissues/organs (Supplementary Fig. S2B), which is similar to the expression profile of the *SOAR1* gene (Mei *et al.*, 2014). Further, we performed BiFC assay in tobacco (*N. benthamiana*) leaves to examine the subcellular compartment in which the interaction of USB1 with SOAR1 takes place, and showed that USB1 localizes to and interacts with SOAR1 in both the nucleus and the cytoplasm (Fig. 1G). This is consistent with the nuclear–cytoplasmic co-localization of these two proteins (Fig. 1F).

### Knockout of *USB1* increases, but its overexpression reduces, ABA sensitivity in post-germination growth

We obtained a mutant *usb1-1* line (SAIL_717_G03), which contains a T-DNA insertion in the first exon as a knockdown allele (Fig. 2A), as evidenced by qRT-PCR (Fig. 2B). Further, we selected a single guide RNA target C1 in the *USB1* gene to generate the *usb1-2* mutant with the CRISPR/Cas9 system (Z.P. Wang *et al.*, 2015). The *usb1-2* mutant has an adenosine insertion in the third exon of the *USB1* gene locus, which generates a premature stop codon and thus would lead to truncated proteins due to a coding frame shift (Fig. 2C).

We investigated ABA sensitivity of the different genotypes in early seedling growth with the seeds sown either directly in ABA-containing medium or in ABA-free medium and then transferred to ABA-containing medium 60 h after stratification. With these two methods we consistently observed that the *usb1-1* and *usb1-2* mutants showed an ABA-hypersensitive phenotype (Fig. 2D, E; Supplementary Figs S3, S4), while the *USB1* overexpression lines displayed an ABA-insensitive phenotype compared with wild-type Col-0 plants (Fig. 2F, G; Supplementary Figs S3, S4) in ABA-induced post-germination growth arrest. It is noteworthy that the early seedling growth of the *usb1-1* and *usb1-2* mutants was significantly reduced compared with wild-type Col-0 plants in the ABA-free medium (Fig. 2D, E; Supplementary Fig S4), suggesting that these mutants are hypersensitive to physiological levels of endogenous ABA. Also, we estimated the effect of ABA on seedling growth with relative growth values based on the absolute values (Fig. 2; Supplementary Figs S3, S4, S8). The ABA-hypersensitive phenotype in the *usb1-1* and *usb1-2* mutants was rescued by introduction of the *USB1*-coding sequence into the *usb1-1* and *usb1-2* plants (Supplementary Fig. S5), demonstrating that the ABA-hypersensitive phenotype was caused by the disruption of the *USB1* gene in these mutants. These data reveal that USB1 negatively regulates ABA signaling in early seedling growth.

Additionally, we observed that the two *usb1* mutant alleles showed hypersensitive phenotypes to both NaCl and mannitol treatments, but the *USB1*overexpression lines displayed insensitive phenotypes in early seedling growth (Supplementary Figs S6, S7). It is noteworthy that ABA hypersensitivity usually enhances tolerance to salt and osmotic stress-induced grown inhibition, whereas ABA insensitivity reduces it. However, in the early post-germination stage, we observed opposite phenotypes, that is, these mutants show similar sensitivities to salt and osmotic stresses as to ABA. This could be attributed to alteration of ABA sensitivity in these mutants under the salt- and osmotically induced high concentrations of ABA (Zhu, 2002; Shinozaki *et al.*, 2003).

### USB1 and SOAR1 function synergistically and are both required in ABA-induced post-germination growth arrest

**Fig. 3.**
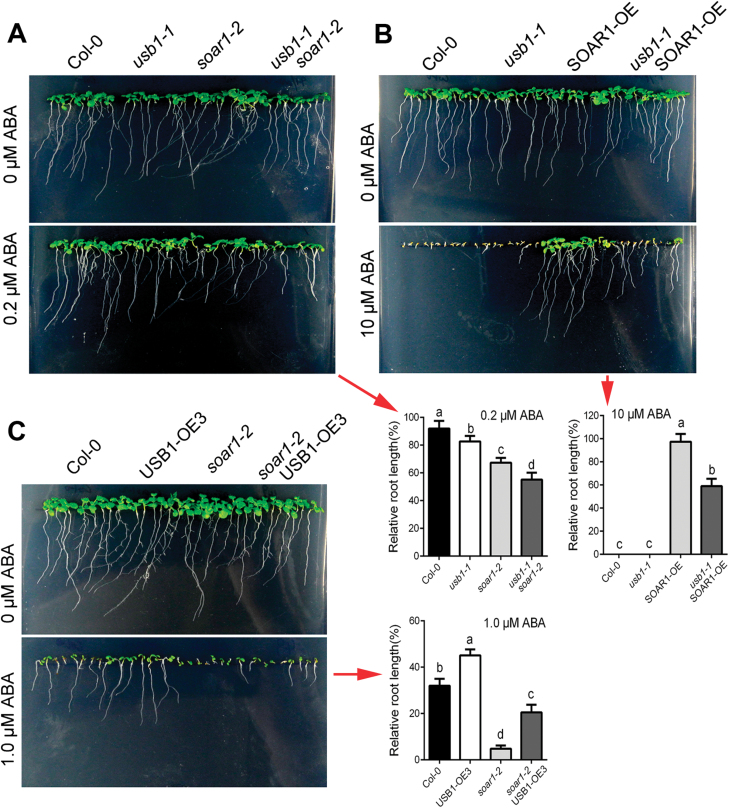
USB1 functionally interacts with SOAR1 in ABA-induced early seedling growth inhibition. (A) Seedling growth of the wild-type Col-0, *usb1-1* and *soar1-2* single mutants, and the *usb1-1 soar1-2* double mutant in ABA-free (0 μΜ) and ABA-containing (0.2 μΜ) medium 10 d after stratification at 4 °C for 3 d. (B) Seedling growth of wild-type Col-0, *usb1-1*, the *SOAR1* overexpression line OE6 (SOAR1-OE), and SOAR1-OE under the *usb1-1* background(*usb1-1* SOAR1-OE) in ABA-free (0 μM) and ABA-containing (10μM) medium. (C) Seedling growth of wild-type Col-0, *soar1-2*, and USB1-OE3 under the *soar1-2* background (*soar1-2* USB1-OE3) in ABA-free (0 μM) and ABA-containing (1.0 μM) medium. In (A–C), the histograms (indicated by red arrows) show statistical analysis of the relative root length of the corresponding different genotypes, and the relative root length of each genotype grown on ABA-containing MS medium was normalized relative to the value of the corresponding genotype at 0 μΜ ABA, which was taken as 100%. Each value is the mean ±SE of five biological determinations, and different letters indicate significant differences at *P*<0.05 (Duncan’s multiple range test) when comparing values within the same ABA concentration.

We previously showed that knockout of *SOAR1* in the *soar1-2* and *soar1-3* mutants results in ABA-hypersensitive phenotypes, and overexpression of *SOAR1* almost completely abolishes ABA-induced inhibition of seed germination and post-germination growth arrest (Jiang *et al*., 2014, 2015; Mei *et al.*, 2014). We therefore tested whether USB1 genetically interacts with SOAR1 in ABA signaling. We observed that the *usb1-1 soar1-2* and *usb1-2 soar1-2* double mutants displayed a stronger ABA-hypersensitive phenotype than their corresponding single mutants*usb1-1*, *usb1-2*, and *soar1-2* (Fig. 3A; Supplementary Fig. S8A, B), indicating that USB1 functions synergistically with SOAR1 in ABA signaling.

Further, we observed that introduction of the *usb1-1* or *usb1-2* mutant allele into the *SOAR* overexpression line OE6 (SOAR1-OE6) partly suppressed the strong ABA-insensitive phenotype of SOAR1-OE6 in early seedling growth (Fig. 3B; Supplementary Fig. S8C, D), revealing that the function of SOAR1 in ABA signaling requires USB1. Similarly, we showed that introduction of the *soar1-2* mutation into the *USB1*overexpression line OE3 (USB1-OE3) partly suppressed the ABA-insensitive phenotype of USB1-OE3 in early seedling growth (Fig. 3C), indicating that the role of USB1 in ABA signaling also depends on SOAR1. Taken together, these data reveal that USB1 and SOAR1 function interdependently and cooperatively in ABA-induced post-germination growth arrest.

### Knockout of *USB1* or *SOAR1* results in a wide range of pre-mRNA splicing defects, and double mutation of the two genes increases the defects

**Fig. 4.**
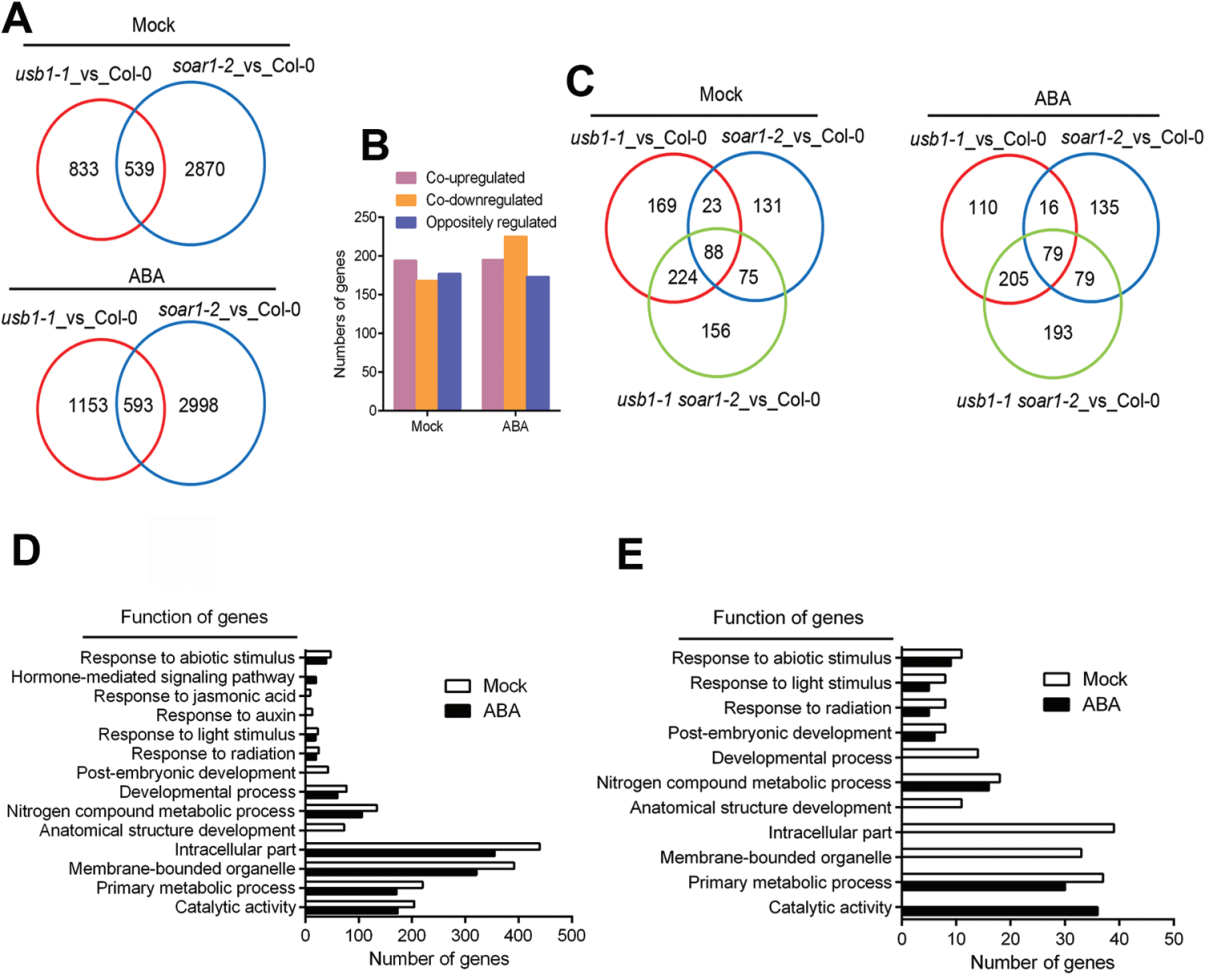
USB1 and SOAR1 co-regulate pre-mRNA splicing of a set of development-/environment-responsive genes. (A) Venn diagram showing the overlaps of genes co-regulated by USB1 and SOAR1 as determined by RNA-seq experiments in the *usb1-1* and *soar1-2* mutants in comparison with the wild-type Col-0 under mock (top) and ABA treatment (bottom) conditions. (B) Histogram showing the numbers of genes co-up-regulated, co-down-regulated, and oppositely regulated by USB1 and SOAR1. (C) Venn diagrams showing the alternative splicing genes in the *usb1-1*, *soar1-2* single mutants, and *usb1-1 soar1-2* double mutant in comparison with the wild-type Col-0 under mock (left) and ABA treatment (right) conditions. (D) Functions of alternative splicing genes regulated by USB1 under mock and ABA treatment conditions. (E) Functions of alternative splicing genes co-regulated by USB1 and SOAR1 under mock and ABA treatment conditions.

We performed high-throughput RNA-seq analysis to clarify the expression profiles regulated by USB1 and SOAR1. The data revealed that the expression levels of 1372 genes (833 + 539) in *usb1-1*, and 3409 genes (2870 + 539) in *soar1-2* were changed (|fold change|>1.5, *P*<0.05) in comparison with wild-type plants without ABA treatment, where expression of 539 genes was potentially co-regulated by USB1 and SOAR1 (Fig. 4A). ABA treatment enhanced the number of genes (1746 genes in *usb1-1*, 3591 genes in *soar1-2*) whose expression levels were altered, with 593 genes potentially co-regulated by USB1 and SOAR1 (Fig. 4A). It is noteworthy that USB1 and SOAR1 oppositely affected a set of genes, and notably cooperated to up- or down-regulate their expression levels. Moreover, ABA treatment increased the number of down-regulated genes (Fig. 4B).

We further analyzed the differential alternative splicing events from RNA-seq data, and found 576 alternative splicing events for 526 genes in the *usb1-1* mutant, while 359 alternative splicing events were found for 334 genes in *soar1-2* compared with wild-type plants without ABA treatment. ABA treatment probably reduced alternative splicing events, with 466 alternative splicing events in 426 genes in *usb1-1*, and 353 alternative splicing events in 326 genes in *soar1-2* (Fig. 4C; Supplementary Fig. S9). It is particularly noteworthy that the total number of splicing events and involved genes increased in the *usb1-1 soar1-2* double mutant compared with that in the *usb1-1* or *soar1-2* single mutant (Fig. 4C; Supplementary Fig. S9). Additionally, we observed that intron retention is the most abundant alternative splicing category amongst the five categories of alternative splicing events: intron retention, exon skipping, alternative 5' SS, alternative 3' SS, and mutually exclusive exon (Supplementary Fig. S9).

GO analysis of the alternative splicing genes, which are regulated by USB1 (Fig. 4D) and co-regulated by both USB1 and SOAR1 (Fig. 4E; Supplementary Fig. S10; Supplementary Table S2), revealed that these genes were involved in many biological processes, such as the response to abiotic stimulus, light stimulus, hormone responses, developmental process, and primary metabolic processes. This implies that USB1 and SOAR1 cooperate to modulate a set of biological processes by regulating pre-mRNA splicing.

### Knockdown of *USB1* or *SOAR1* affects accurate mRNA splicing of a subset of ABA-responsive genes, and double mutation of the two genes enhances the defects

**Fig. 5.**
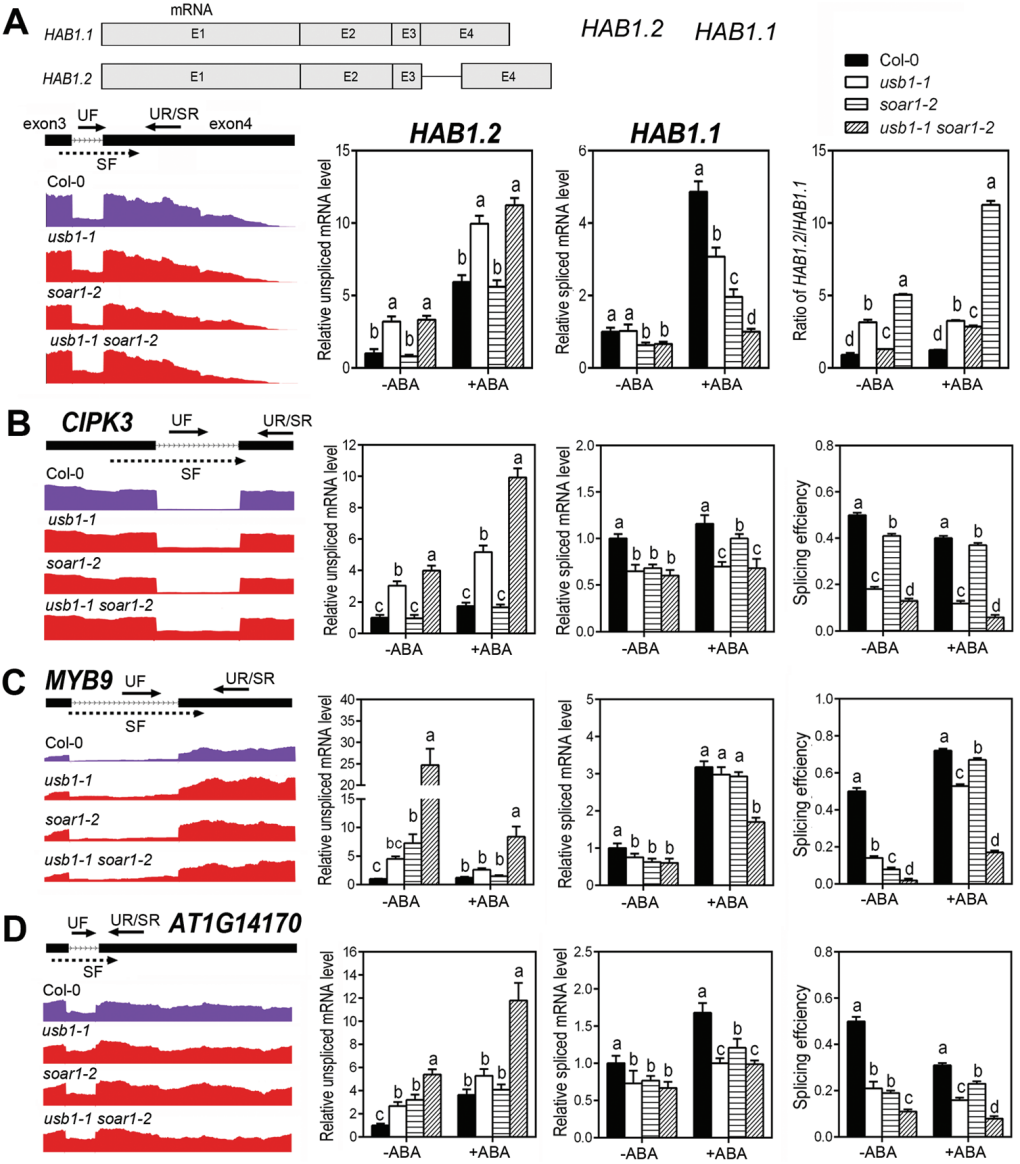
USB1 and SOAR1 cooperate to regulate the alternative splicing of a subset of genes involved in ABA/stress signaling. (A) Top: diagrams of the *HAB1.1* (also called spliced mRNA) and *HAB1.2* (also called unspliced mRNA) variants. Boxes and lines represent exons and introns, respectively (figure drawn to scale). E1–E4 represent exons 1–4. Bottom left panel: the intron retention events of *HAB1*, visualized by Rmats2sashimiplot in the wild-type Col-0, *usb1-1* and *soar1-2* single mutants, and *usb1-1 soar1-2* double mutant without ABA treatment. Bottom right panel: the *HAB1* variants in the different genotypes as described in the left panel, identified by qRT-PCR under ABA-free (–ABA) or ABA treatment (+ABA). Primers for detecting *HAB1.1* (spliced mRNA) and *HAB1.2* (unspliced mRNA) transcripts are shown in the diagram in the left panel, in which the dashed line indicates the intron, and the black/bold line the exon. UF represents the unspliced forward primer and UR the unspliced reverse primer for detecting the unspliced mRNA level, which are designed according to the unspliced intron and adjacent exon, respectively (Supplementary Table S1). SF, spliced forward primer, and SR, spliced reverse primer for detecting the spliced mRNA level, are designed according to the sequences across exon–exon junctions, in which the reverse primer SR is the same as UR. (B–D) The intron retention events of *CIPK3* (retention of intron 12, B), *MYB9* (retention of intron 1, C), and *AT1G14170* (retention of intron 1, D), visualized by the Rmats2sashimiplot (left, color panels) and identified by qRT-PCR under ABA-free (–ABA) or ABA treatment (+ABA) (right, column panels) in Col-0, *usb1-1*and *soar1-2* single mutants, and the *usb1-1 soar1-2* double mutant. The primers UF, UR, SF, and SR for detecting spliced and unspliced mRNA transcripts, shown in the corresponding diagram, are the same as described in (A). Splicing efficiency of each genotype (right column) is referred to as the ratio of the level of spliced mRNA relative to the level of total mRNA (spliced plus unspliced mRNA). Each value is the mean ±SE of five biological determinations, and different letters indicate significant differences at *P*<0.05 (Duncan’s multiple range test) when comparing values of the different genotypes within the same treatment (–ABA, ABA-free; and +ABA, ABA treatment).

RNA-seq analysis showed the intron retention of several ABA-responsive genes in *usb1-1* and *soar1-2* mutants, such as *HAB1*, *CIPK3*, *MYB9*, and *AT1G14170*. *HAB1* (At1g72770) encodes a member of the PP2Cs negatively involved in ABA signaling (Umezawa *et al.*, 2009; Vlad *et al.*, 2009). *HAB1* transcripts have four variants,*HAB1.1*, *HAB1.2*, *HAB1.3*, and *HAB1.4*, in which retention of the third intron (unspliced) mRNA transcript was regarded as *HAB1.2* and spliced mRNA as *HAB1.1*(Z. Wang *et al.*, 2015; Zhan *et al.*, 2015). We showed that the levels of *HAB1.1* and *HAB1.2* were altered in the *usb1-1* and *soar1-2* single and *usb1-1 soar1-2* double mutants compared with wild-type plants Fig. 5A; Supplementary Fig. S11). This results in a significantly increased ratio of *HAB1.2* to *HAB1.1* in these mutants, especially in the *usb1-1 soar1-2* double mutant in comparison with wild-type plants, and ABA treatment amplified the differences in the ratio of *HAB1.2* to *HAB1.1* among these genotypes (Fig. 5A; Supplementary Fig. S11).

The *CIPK3* gene (At2g26980) encodes a calcineurin B-like-interacting protein kinase 3 that negatively regulates ABA signaling (Kim *et al.*, 2003; Pandey *et al.*, 2008; Sanyal *et al.*, 2017*a*, *b*). *MYB9*(At5g16770) encodes an ABA-responsive transcription factor (Chen *et al.*, 2006). The At1g14170 locus encodes an RNA-binding protein, whose phosphorylation level is repressed by ABA (Kline *et al.*, 2010). We showed that the levels of alternative splicing events for these genes were significantly altered in the *usb1-1* and *soar1-2* single and *usb1-1 soar1-2* double mutants compared with wild-type plants, particularly with the most marked change in the *usb1-1 soar1-2* double mutant, which results in a significant decrease of the mature mRNA levels (relative spliced mRNA levels) and splicing efficiency (Fig. 5B–D; Supplementary Fig. S12). It is noteworthy that ABA treatment significantly affected these splicing events compared with control treatment (Fig. 5B–D). Taken together, these findings indicate that both USB1 and SOAR1 are required for the accurate mRNA splicing of the ABA-responsive genes.

Given that ABI5 was previously shown to be a downstream player of ABAR–SOAR1 coupled signaling (Mei *et al.*, 2014) and that the *ABI5* mRNA is a target of the mRNA cap-binding complex in SOAR1–eIFiso4G1/2 coupled signaling (Bi *et al.*, 2019), we tested if *ABI5* expression is altered in the *usb1-1* and *usb1-2* mutants. We found that loss-of-function of *USB1* did not affect *ABI5* expression (Supplementary Fig. S13), suggesting that *ABI5* may not be a target of USB1 in this pre-mRNA splicing event.

### Loss-of-function of *USB1* reduces, but overexpression of *USB1* improves plant growth and development

Previous studies in Arabidopsis and maize showed that U6 snRNP plays a crucial role in plant development throughout the life cycle. The Arabidopsis LSM2–LSM8 complex is an essential component of the U6 snRNP and LSM1- and LSM8-deficient mutants display severe developmental alterations (Perea-Resa *et al.*, 2012). Loss-of-function mutation of the maize *USB1* gene affects seed development (Li *et al.*, 2017). Given that Arabidopsis USB1 and SOAR1 cooperate to regulate a wide range of pre-mRNA splicing involved in diverse developmental processes as mentioned above (Fig. 4; Supplementary Figs. S9, S10; Supplementary Table S2), we investigated physiological consequences caused by loss-of-function or overexpression of *USB1* during the life cycle of these plants. We observed that loss-of-function of *USB1* reduced, but overexpression of *USB1* enhanced, plant growth, as estimated by both height and weight, silique length and number, and seed size and total weight (Supplementary Fig. S13). Thus, loss-of-function of *USB1* severely affects, but overexpression of *USB1* improves plant growth and development, suggesting the potential use of the *USB1* gene in crop improvement. These findings, consistent with previous observations (Perea-Resa *et al.*, 2012), indicate the crucial roles of U6 snRNP in the whole developmental process.

## Discussion

### USB1 directly interacts with SOAR1 to negatively regulate ABA signaling

As a 3'–5' exoribonuclease that potentially generates U6 snRNA for mRNA spliceosome assembly, USB1 was shown to regulate seed development in maize (Li *et al.*, 2017), but the function of Arabidopsis USB1 remained unknown. In the present study, we showed that USB1 is an interaction partner of SOAR1 (Fig. 1). Knockout of the *USB1* gene increases ABA sensitivity in early seedling growth (Fig 2) but overexpression reduces it, demonstrating that USB1 functions negatively in ABA signaling. This negative role of USB1 is consistent with that of its interaction partner SOAR1, which functions as a crucial, negative player of ABA signaling (Jiang *et al*., 2014, 2015; Mei *et al.*, 2014). Further, we showed that the *usb1-1 soar1-2* and *usb1-2 soar1-2* double mutants displayed additive ABA-hypersensitive phenotypes compared with either single mutant alone (Fig. 3), indicating that USB1 functions synergistically with SOAR1 in ABA signaling. This suggests that USB1 functions in either an independent/parallel pathway from SOAR1 or in the same pathway. It is particularly noteworthy that the *soar1* null allele is lethal and *soar1-2* is a knockdown allele (Mei *et al.*, 2014). The additive ABA-hypersensitive phenotypes of the *usb1 soar1-2* double mutants suggest that USB1 and SOAR1 may function in the same pathway rather than in a parallel pathway, given that adding together two hypomorphic loss-of-function mutations in genes within the same pathway would be expected to lead to additive phenotypes. Further genetic findings revealed that USB1 and SOAR1 are mutually required to function cooperatively in ABA-induced post-germination growth arrest (Fig. 3), supporting the idea that USB1 functions in the same pathway as SOAR1 and at the same signaling node.

### USB1 and SOAR1 cooperatively regulate pre-mRNA splicing involved in ABA signaling

Alternative pre-mRNA splicing was reported to be involved in plant responses to ABA, high salinity, extreme temperatures, and drought stress (Xiong *et al.*,2001; Zhang *et al.*, 2011; Cui *et al.*, 2014; Kong *et al.*, 2014; Z. Wang *et al.*, 2015; Zhan *et al.*, 2015; Carrasco-Lopez *et al*., 2017; Kim *et al.*,2017). However, the mechanism by which ABA signaling affects the alternative splicing process remains largely unknown. In the present study, we showed a potential mechanism involving both USB1 and SOAR1. Previous reports showed that USB1 is involved in the pre-mRNA splicing events in yeast and maize, where loss-of-function of the *USB1* gene leads to pre-mRNA splicing defects (Shchepachev *et al.*, 2012; Li *et al.*,2017). Consistent with these previous findings, we observed that underexpression of *USB1* or *SOAR1* results in a wide range of pre-mRNA splicing defects in which the mRNA splicing of a subset of ABA-responsive genes was affected, and double mutation of *USB1* and *SOAR1* genes enhanced the defects (Figs 4, 5; Supplementary FigsS9–12; Supplementary Table S2). These findings indicate that both USB1 and SOAR1 are required for accurate mRNA splicing of ABA-responsive genes.

 It is particularly noteworthy that underexpression of USB1 or SOAR1 alters pre-mRNA splicing of a member of the clade-A PP2Cs, which negatively regulate ABA signaling by dephosphorylating SnRK2 protein kinases, especially SnRK2.6 (Umezawa *et al.*, 2009; Vlad *et al.*, 2009). The *HAB1* pre-mRNA undergoes alternative splicing to produce two functional splice variants *HAB1.1* and *HAB1.2*. *HAB1.2* is shorter than *HAB1.1* because of retention of the third intron generates a premature stop codon. HAB1.2 protein can interact with SnRK2.6 but fails to dephosphorylate OST1 kinase activity, probably by which HAB1.2 positively regulates ABA signaling. Importantly, the ratio of *HAB1.2* to *HAB1.1* alternative splice forms was shown to be an on and off switch in ABA signal transduction (Z. Wang *et al.*, 2015; Zhan *et al.*, 2015). We showed that knockout of USB1 or SOAR1 in the *usb1-1* and *soar1-2* single mutants increased the ratio of *HAB1.2* to *HAB1.1*, with amplified effects in the *usb1-1 soar1-2* double mutant, especially after ABA treatment (Fig. 5A; Supplementary Fig. S11). This suggests that USB1- and SOAR1-coupled signaling, together with the previously reported function of a homolog of human splicing factor RBM25, ROA1/RBM25 (Z. Wang *et al.*, 2015; Zhan *et al.*, 2015), regulates alternative splicing of *HAB1* to participate in ABA signaling. The higher ratio of *HAB1.2* to *HAB1.1* explains, at least partly, the ABA-hypersensitive phenotype of*usb1-1* and *soar1-2* single and *usb1-1 soar1-2* double mutants (Figs 2, 3; Supplementary Figs S3–S8).

Previous reports showed that the *CIPK3* transcript has five splice variants, namely*CIPK3.1*, *CIPK3.2*, *CIPK3.3*, *CIPK3.4*, and *CIPK3.5*, among which only *CIPK3.4* is a functional transcript accurately spliced with all 14 introns deleted (Sanyal *et al.*, 2017*a*). We found that the splicing efficiency of *CIPK3* and the level of the mature *CIPK3* mRNA decreased in the *usb1-1* and *usb1-1 soar1-2* mutants (Fig. 5B; Supplementary Fig. S12), indicating that USB1/SOAR1-coupled signaling positively regulates splicing of functional *CIPK3* mRNA coding for a negative regulator of ABA signaling (Kim *et al.*, 2003; Pandey *et al.*, 2008). This is consistent with the ABA-hypersensitive phenotype in *usb1-1* single and *usb1-1 soar1-2* double mutants (Figs 2, 3; Supplementary Figs S3–8). USB1 and SOAR1 also regulated mRNA splicing of the ABA-responsive transcription factor MYB9 (Chen *et al.*, 2006) and the ABA-responsive RNA-binding protein encoded by the At1g14170 locus (Kline *et al.*, 2010) (Fig. 5; Supplementary Fig. S12), suggesting that USB1 and SOAR1 also play important regulatory roles for these modulators of ABA signaling.

 Previous studies showed that the essential components of the Arabidopsis U6 snRNP, LSM4/5/8, regulate pre-mRNA splicing of stress-responsive genes to modulate plant responses to environmental stresses (Zhang *et al.*, 2011; Cui *et al.*, 2014; Carrasco-Lopez *et al*., 2017). Importantly, Carrasco-Lopez *et al*. (2017) revealed that the Arabidopsis LSM2–LSM8 complex regulates spliceosome activity specifically in response to changing environmental conditions, ensuring the efficiency and accuracy of constitutive and alternative splicing of selected pre-mRNAs, and thus adequate plant adaptation to abiotic stresses (Carrasco-Lopez *et al*., 2017). USB1- and SOAR1-coupled ABA signaling may function in a similar manner, ensuring an adequate ABA response to balance plant development and stress tolerance, but the regulatory details of this process will require further research.

 We previously reported that SOAR1 is a unique cytoplasmic–nuclear dual-localized PPR protein negatively involved in ABA signaling (Mei *et al.*, 2014; Jiang *et al.*, 2014, 2015) partly by affecting assembly of the mRNA cap-binding complex to affect mRNA translation (Bi *et al.*, 2019). The present study reveals that SOAR1 interacts with USB1 to regulate ABA signaling probably by regulating assembly of the mRNA spliceosome to modulate pre-mRNA splicing of ABA-responsive genes. These findings provide a new link of ABA signaling with the pre-mRNA splicing process, and help to understand complex ABA signaling pathways.

## Supplementary data

The following supplementary data are available at *JXB* online.

**Fig. S1.** Phylogenetic analysis of Arabidopsis USB1 protein homologs.

**Fig. S2.** Expression profile of the *USB1* gene and subcellular localization of USB1 protein.

**Fig. S3.** Early seedling growth of the *usb1* mutants under higher concentrations of ABA.

**Fig. S4.** Early seedling growth of the *usb1* mutants and *USB1* overexpression lines, assayed by transferring germinating seeds to ABA-containing medium.

**Fig. S5.** Phenotypic analysis of the complementation lines of the *usb1* mutants.

**Fig. S6.** Early seedling growth of different genotypes under salt stress.

**Fig. S7.** Early seedling growth of different genotypes under d-mannitol-induced osmotic stress.

**Fig. S8.** USB1 functionally interacts with SOAR1 in ABA-induced early seedling growth inhibition.

**Fig. S9.** Quantification of alternative splicing events.

**Fig. S10.**Gene Ontology analysis of genes co-regulated by USB1 and SOAR1 under mock and ABA treatment conditions.

**Fig. S11.** The intron retention events of *HAB1* in the different genotypes.

**Fig. S12.** Diagrams of the intron retention events of *CIPK3*, *MYB9*, and *AT1G14170* in the different genotypes under mock and ABA treatment conditions.

**Fig. S13.** Loss-of-function of *USB1* does not affect *ABI5* expression.

**Fig. S14.** Phenotypic observations of *usb1* mutants and *USB1* overexpression lines during the plants’ life cycle .

**Table S1.** Primers used in this study.

**Table S2.** RNA-seq data: function of genes co-regulated by USB1 and SOAR1.

eraa315_suppl_Supplementary_Material

## References

[CIT0001] Adie BA, Pérez-Pérez J, Pérez-Pérez MM, Godoy M, Sánchez-Serrano JJ, Schmelz EA, Solano R 2007 ABA is an essential signal for plant resistance to pathogens affecting JA biosynthesis and the activation of defenses in *Arabidopsis*. The Plant Cell 19, 1665–1681. 17513501 10.1105/tpc.106.048041 PMC1913739

[CIT0002] Bi C, Ma Y, Jiang SC, Mei C, Wang XF, Zhang DP 2019 *Arabidopsis* translation initiation factors eIFiso4G1/2 link repression of mRNA cap-binding complex eIFiso4F assembly with RNA-binding protein SOAR1-mediated ABA signaling. New Phytologist 223, 1388–1406. 31050354 10.1111/nph.15880

[CIT0003] Brzyżek G, Świeżewski S 2015 Mutual interdependence of splicing and transcription elongation. Transcription 6, 37–39. 25890997 10.1080/21541264.2015.1040146 PMC4581361

[CIT0004] Carrasco-López C, Hernández-Verdeja T, Perea-Resa C, Abia D, Catalá R, Salinas J 2017 Environment-dependent regulation of spliceosome activity by the LSM2–8 complex in *Arabidopsis*. Nucleic Acids Research 45, 7416–7431. 28482101 10.1093/nar/gkx375 PMC5499552

[CIT0005] Chen YH, Yang XY, He K, et al 2006 The MYB transcription factor superfamily of *Arabidopsis*: expression analysis and phylogenetic comparison with the rice MYB family. Plant Molecular Biology 60, 107–124. 16463103 10.1007/s11103-005-2910-y

[CIT0006] Cui P, Zhang S, Ding F, Ali S, Xiong L 2014 Dynamic regulation of genome-wide pre-mRNA splicing and stress tolerance by the Sm-like protein LSm5 in *Arabidopsis*. Genome Biology 15, R1. 24393432 10.1186/gb-2014-15-1-r1 PMC4053965

[CIT0007] Cutler SR, Rodriguez PL, Finkelstein RR, Abrams SR 2010 Abscisic acid: emergence of a core signaling network. Annual Review of Plant Biology 61, 651–679. 10.1146/annurev-arplant-042809-112122 20192755

[CIT0008] Didychuk AL, Montemayor EJ, Brow DA, Butcher SE 2016 Structural requirements for protein-catalyzed annealing of U4 and U6 RNAs during di-snRNP assembly. Nucleic Acids Research 44, 1398–1410. 26673715 10.1093/nar/gkv1374 PMC4756825

[CIT0009] Didychuk AL, Montemayor EJ, Carrocci TJ, DeLaitsch AT, Lucarelli SE, Westler WM, Brow DA, Hoskins AA, Butcher SE 2017 Usb1 controls U6 snRNP assembly through evolutionarily divergent cyclic phosphodiesterase activities. Nature Communications 8, 497. 10.1038/s41467-017-00484-w PMC5591277 28887445

[CIT0010] Dobin A, Davis CA, Schlesinger F, Drenkow J, Zaleski C, Jha S, Batut P, Chaisson M, Gingeras TR 2013 STAR: ultrafast universal RNA-seq aligner. Bioinformatics 29, 15–21. 23104886 10.1093/bioinformatics/bts635 PMC3530905

[CIT0011] Fairchild CD, Schumaker MA, Quail PH 2000 HFR1 encodes an atypical bHLH protein that acts in phytochrome A signal transduction. Genes & Development 14, 2377–2391. 10995393 PMC316929

[CIT0012] Fica SM, Nagai K 2017 Cryo-electron microscopy snapshots of the spliceosome: structural insights into a dynamic ribonucleoprotein machine. Nature Structural & Molecular Biology 24, 791–799. 10.1038/nsmb.3463 PMC6386135 28981077

[CIT0013] Finkelstein RR, Gampala SS, Rock CD 2002 Abscisic acid signaling in seeds and seedlings. The Plant Cell 14 Suppl, S15–S45. 12045268 10.1105/tpc.010441 PMC151246

[CIT0014] Halbeisen RE, Galgano A, Scherrer T, Gerber AP 2008 Post-transcriptional gene regulation: from genome-wide studies to principles. Cellular and Molecular Life Sciences 65, 798–813. 18043867 10.1007/s00018-007-7447-6 PMC2771128

[CIT0015] Hilcenko C, Simpson PJ, Finch AJ, et al 2013 Aberrant 3' oligoadenylation of spliceosomal U6 small nuclear RNA in poikiloderma with neutropenia. Blood 121, 1028–1038. 23190533 10.1182/blood-2012-10-461491

[CIT0016] Huertas R, Catalá R, Jiménez-Gómez JM, Mar Castellano M, Crevillén P, Piñeiro M, Jarillo JA, Salinas J 2019 Arabidopsis SME1 regulates plant development and response to abiotic stress by determining spliceosome activity specificity. The Plant Cell 31, 537–554. 30696706 10.1105/tpc.18.00689 PMC6447010

[CIT0017] Hugouvieux V, Kwak JM, Schroeder JI 2001 An mRNA cap binding protein, ABH1, modulates early abscisic acid signal transduction in *Arabidopsis*. Cell 106, 477–487. 11525733 10.1016/s0092-8674(01)00460-3

[CIT0018] Iwata H, Gotoh O 2011 Comparative analysis of information contents relevant to recognition of introns in many species. BMC Genomics 12, 45. 21247441 10.1186/1471-2164-12-45 PMC3033335

[CIT0019] Jang IC, Yang JY, Seo HS, Chua NH 2005 HFR1 is targeted by COP1 E3 ligase for post-translational proteolysis during phytochrome A signaling. Genes & Development 19, 593–602. 15741320 10.1101/gad.1247205 PMC551579

[CIT0020] Jia Q, Liu N, Xie K, et al 2016 CLCuMuB βC1 subverts ubiquitination by interacting with NbSKP1s to enhance geminivirus infection in *Nicotiana benthamiana*. PLoS Pathogens 12, e1005668. 27315204 10.1371/journal.ppat.1005668 PMC4912122

[CIT0021] Jiang SC, Mei C, Liang S, Yu YT, Lu K, Wu Z, Wang XF, Zhang DP 2015 Crucial roles of the pentatricopeptide repeat protein SOAR1 in Arabidopsis response to drought, salt and cold stresses. Plant Molecular Biology 88, 369–385. 26093896 10.1007/s11103-015-0327-9 PMC4486114

[CIT0022] Jiang SC, Mei C, Wang XF, Zhang DP 2014 A hub for ABA signaling to the nucleus: significance of a cytosolic and nuclear dual-localized PPR protein SOAR1 acting downstream of Mg-chelatase H subunit. Plant Signaling & Behavior 9, e972899. 25482771 10.4161/15592316.2014.972899 PMC5155504

[CIT0023] Jurica MS, Moore MJ 2003 Pre-mRNA splicing: awash in a sea of proteins. Molecular Cell 12, 5–14. 12887888 10.1016/s1097-2765(03)00270-3

[CIT0024] Kim GD, Cho YH, Lee BH, Yoo SD 2017 STABILIZED1 modulates pre-mRNA splicing for thermotolerance. Plant Physiology 173, 2370–2382. 28223317 10.1104/pp.16.01928 PMC5373063

[CIT0025] Kim KN, Cheong YH, Grant JJ, Pandey GK, Luan S 2003 CIPK3, a calcium sensor-associated protein kinase that regulates abscisic acid and cold signal transduction in *Arabidopsis*. The Plant Cell 15, 411–423. 12566581 10.1105/tpc.006858 PMC141210

[CIT0026] Kline KG, Barrett-Wilt GA, Sussman MR 2010 In planta changes in protein phosphorylation induced by the plant hormone abscisic acid. Proceedings of the National Academy of Sciences, USA 107, 15986–15991. 10.1073/pnas.1007879107 PMC2936636 20733066

[CIT0027] Koncz C, Dejong F, Villacorta N, Szakonyi D, Koncz Z 2012 The spliceosome-activating complex: molecular mechanisms underlying the function of a pleiotropic regulator. Frontiers in Plant Science 3, 9. 22639636 10.3389/fpls.2012.00009 PMC3355604

[CIT0028] Kong X, Ma L, Yang L, Chen Q, Xiang N, Yang Y, Hu X 2014 Quantitative proteomics analysis reveals that the nuclear cap-binding complex proteins *Arabidopsis* CBP20 and CBP80 modulate the salt stress response. Journal of Proteome Research 13, 2495–2510. 24689873 10.1021/pr4012624

[CIT0029] Laloum T, Martín G, Duque P 2018 Alternative splicing control of abiotic stress responses. Trends in Plant Science 23, 140–150. 29074233 10.1016/j.tplants.2017.09.019

[CIT0030] Li J, Fu J, Chen Y, et al 2017 The U6 biogenesis-like 1 plays an important role in maize kernel and seedling development by affecting the 3' end processing of U6 snRNA. Molecular Plant 10, 470–482. 27825944 10.1016/j.molp.2016.10.016

[CIT0031] Liao Y, Smyth GK, Shi W 2014 featureCounts: an efficient general purpose program for assigning sequence reads to genomic features. Bioinformatics 30, 923–930. 24227677 10.1093/bioinformatics/btt656

[CIT0032] Licht K, Medenbach J, Lührmann R, Kambach C, Bindereif A 2008 3'-Cyclic phosphorylation of U6 snRNA leads to recruitment of recycling factor p110 through LSm proteins. RNA 14, 1532–1538. 18567812 10.1261/rna.1129608 PMC2491463

[CIT0033] Luco RF, Allo M, Schor IE, Kornblihtt AR, Misteli T 2011 Epigenetics in alternative pre-mRNA splicing. Cell 144, 16–26. 21215366 10.1016/j.cell.2010.11.056 PMC3038581

[CIT0034] Marquez Y, Brown JW, Simpson C, Barta A, Kalyna M 2012 Transcriptome survey reveals increased complexity of the alternative splicing landscape in *Arabidopsis*. Genome Research 22, 1184–1195. 22391557 10.1101/gr.134106.111 PMC3371709

[CIT0035] Martin M 2011 Cutadapt removes adapter sequences from high-throughput sequencing reads. EMBnet Journal 17, 10–12.

[CIT0036] Mei C, Jiang SC, Lu YF, et al 2014 *Arabidopsis* pentatricopeptide repeat protein SOAR1 plays a critical role in abscisic acid signalling. Journal of Experimental Botany 65, 5317–5330. 25005137 10.1093/jxb/eru293 PMC4157714

[CIT0037] Mroczek S, Krwawicz J, Kutner J, Lazniewski M, Kuciński I, Ginalski K, Dziembowski A 2012 C16orf57, a gene mutated in poikiloderma with neutropenia, encodes a putative phosphodiesterase responsible for the U6 snRNA 3' end modification. Genes & Development 26, 1911–1925. 22899009 10.1101/gad.193169.112 PMC3435495

[CIT0038] Nieto Moreno N, Giono LE, Cambindo Botto AE, Muñoz MJ, Kornblihtt AR 2015 Chromatin, DNA structure and alternative splicing. FEBS Letters 589, 3370–3378. 26296319 10.1016/j.febslet.2015.08.002

[CIT0039] Pan Q, Shai O, Lee LJ, Frey BJ, Blencowe BJ 2008 Deep surveying of alternative splicing complexity in the human transcriptome by high-throughput sequencing. Nature Genetics 40, 1413–1415. 18978789 10.1038/ng.259

[CIT0040] Pandey GK, Grant JJ, Cheong YH, Kim BG, Li le G, Luan S 2008 Calcineurin-B-like protein CBL9 interacts with target kinase CIPK3 in the regulation of ABA response in seed germination. Molecular Plant 1, 238–248. 19825536 10.1093/mp/ssn003

[CIT0041] Papp I, Mur LA, Dalmadi A, Dulai S, Koncz C 2004 A mutation in the cap binding protein 20 gene confers drought tolerance to Arabidopsis. Plant Molecular Biology 55, 679–686. 15604709 10.1007/s11103-004-1680-2

[CIT0042] Perea-Resa C, Carrasco-López C, Catalá R, Turečkov á V, Novak O, Zhang W, Sieburth L, Jiménez-Gómez JM, Salinas J 2016 The LSM1–7 complex differentially regulates *Arabidopsis* tolerance to abiotic stress conditions by promoting selective mRNA decapping. The Plant Cell 28, 505–520. 26764377 10.1105/tpc.15.00867 PMC4790874

[CIT0043] Perea-Resa C, Hernández-Verdeja T, López-Cobollo R, del Mar Castellano M, Salinas J 2012 LSM proteins provide accurate splicing and decay of selected transcripts to ensure normal *Arabidopsis* development. The Plant Cell 24, 4930–4947. 23221597 10.1105/tpc.112.103697 PMC3556967

[CIT0044] Raczynska KD, Simpson CG, Ciesiolka A, Szewc L, Lewandowska D, McNicol J, Szweykowska-Kulinska Z, Brown JW, Jarmolowski A 2010 Involvement of the nuclear cap-binding protein complex in alternative splicing in *Arabidopsis thaliana*. Nucleic Acids Research 38, 265–278. 19864257 10.1093/nar/gkp869 PMC2800227

[CIT0045] Reddy AS, Marquez Y, Kalyna M, Barta A 2013 Complexity of the alternative splicing landscape in plants. The Plant Cell 25, 3657–3683. 24179125 10.1105/tpc.113.117523 PMC3877793

[CIT0046] Romero-Barrios N, Legascue MF, Benhamed M, Ariel F, Crespi M 2018 Splicing regulation by long noncoding RNAs. Nucleic Acids Research 46, 2169–2184. 29425321 10.1093/nar/gky095 PMC5861421

[CIT0047] Sakharkar MK, Chow VT, Kangueane P 2004 Distributions of exons and introns in the human genome. In Silico Biology 4, 387–393. 15217358

[CIT0048] Sanyal SK, Kanwar P, Samtani H, Kaur K, Jha SK, Pandey GK 2017 Alternative splicing of CIPK3 results in distinct target selection to propagate ABA signaling in *Arabidopsis*. Frontiers in Plant Science 8, 1924. 29225607 10.3389/fpls.2017.01924 PMC5705611

[CIT0049] Sanyal SK, Kanwar P, Yadav AK, Sharma C, Kumar A, Pandey GK 2017 Arabidopsis CBL interacting protein kinase 3 interacts with ABR1, an APETALA2 domain transcription factor, to regulate ABA responses. Plant Science 254, 48–59. 27964784 10.1016/j.plantsci.2016.11.004

[CIT0050] Shang Y, Yan L, Liu ZQ, et al 2010 The Mg-chelatase H subunit of Arabidopsis antagonizes a group of WRKY transcription repressors to relieve ABA-responsive genes of inhibition. The Plant Cell 22, 1909–1935. 20543028 10.1105/tpc.110.073874 PMC2910980

[CIT0051] Shchepachev V, Wischnewski H, Missiaglia E, Soneson C, Azzalin CM 2012 Mpn1, mutated in poikiloderma with neutropenia protein 1, is a conserved 3'-to-5' RNA exonuclease processing U6 small nuclear RNA. Cell Reports 2, 855–865. 23022480 10.1016/j.celrep.2012.08.031

[CIT0052] Shen S, Park JW, Lu ZX, Lin L, Henry MD, Wu YN, Zhou Q, Xing Y 2014 rMATS: robust and flexible detection of differential alternative splicing from replicate RNA-Seq data. Proceedings of the National Academy of Sciences, USA 111, E5593–E5601. 10.1073/pnas.1419161111 PMC4280593 25480548

[CIT0053] Shen YY, Wang XF, Wu FQ, et al 2006 The Mg-chelatase H subunit is an abscisic acid receptor. Nature 443, 823–826. 17051210 10.1038/nature05176

[CIT0054] Shi Y 2017 The spliceosome: a protein-directed metalloribozyme. Journal of Molecular Biology 429, 2640–2653. 28733144 10.1016/j.jmb.2017.07.010

[CIT0055] Shinozaki K, Yamaguchi-Shinozaki K, Seki M 2003 Regulatory network of gene expression in the drought and cold stress responses. Current Opinion in Plant Biology 6, 410–417. 12972040 10.1016/s1369-5266(03)00092-x

[CIT0056] Simpson CG, Thow G, Clark GP, Jennings SN, Watters JA, Brown JW 2002 Mutational analysis of a plant branchpoint and polypyrimidine tract required for constitutive splicing of a mini-exon. RNA 8, 47–56. 11873758 10.1017/s1355838202015546 PMC1370234

[CIT0057] Tian T, Liu Y, Yan H, You Q, Yi X, Du Z, Xu W, Su Z 2017 agriGO v2.0: a GO analysis toolkit for the agricultural community, 2017 update. Nucleic Acids Research 45, W122–W129. 28472432 10.1093/nar/gkx382 PMC5793732

[CIT0058] Umezawa T, Sugiyama N, Mizoguchi M, Hayashi S, Myouga F, Yamaguchi-Shinozaki K, Ishihama Y, Hirayama T, Shinozaki K 2009 Type 2C protein phosphatases directly regulate abscisic acid-activated protein kinases in *Arabidopsis*. Proceedings of the National Academy of Sciences, USA 106, 17588–17593. 10.1073/pnas.0907095106 PMC2754379 19805022

[CIT0059] Vlad F, Rubio S, Rodrigues A, Sirichandra C, Belin C, Robert N, Leung J, Rodriguez PL, Laurière C, Merlot S 2009 Protein phosphatases 2C regulate the activation of the Snf1-related kinase OST1 by abscisic acid in *Arabidopsis*. The Plant Cell 21, 3170–3184. 19855047 10.1105/tpc.109.069179 PMC2782292

[CIT0060] Walter M, Chaban C, Schütze K, et al 2004 Visualization of protein interactions in living plant cells using bimolecular fluorescence complementation. The Plant Journal 40, 428–438. 15469500 10.1111/j.1365-313X.2004.02219.x

[CIT0061] Wang Z, Ji H, Yuan B, Wang S, Su C, Yao B, Zhao H, Li X 2015 ABA signalling is fine-tuned by antagonistic HAB1 variants. Nature Communications 6, 8138. 10.1038/ncomms9138 26419884

[CIT0062] Wang ZP, Xing HL, Dong L, Zhang HY, Han CY, Wang XC, Chen QJ 2015 Egg cell-specific promoter-controlled CRISPR/Cas9 efficiently generates homozygous mutants for multiple target genes in *Arabidopsis* in a single generation. Genome Biology 16, 144. 26193878 10.1186/s13059-015-0715-0 PMC4507317

[CIT0063] Will CL, Luhrmann R 2011 Spliceosome structure and function. Cold Spring Harbor Perspectives in Biology 3, a003707. 21441581 10.1101/cshperspect.a003707 PMC3119917

[CIT0064] Wu FQ, Xin Q, Cao Z, et al 2009 The magnesium-chelatase H subunit binds abscisic acid and functions in abscisic acid signaling: new evidence in Arabidopsis. Plant Physiology 150, 1940–1954. 19535472 10.1104/pp.109.140731 PMC2719140

[CIT0065] Xiong F, Ren JJ, Yu Q, Wang YY, Lu CC, Kong LJ, Otegui MS, Wang XL 2019 AtU2AF65b functions in abscisic acid mediated flowering via regulating the precursor messenger RNA splicing of ABI5 and FLC in *Arabidopsis*. New Phytologist 223, 277–292. 30790290 10.1111/nph.15756

[CIT0066] Xiong L, Gong Z, Rock CD, Subramanian S, Guo Y, Xu W, Galbraith D, Zhu JK 2001 Modulation of abscisic acid signal transduction and biosynthesis by a Sm-like protein in *Arabidopsis*. Development Cell 1, 771–781. 10.1016/s1534-5807(01)00087-9 11740939

[CIT0067] Yoo SD, Cho YH, Sheen J 2007 *Arabidopsis* mesophyll protoplasts: a versatile cell system for transient gene expression analysis. Nature Protocols 2, 1565–1572. 17585298 10.1038/nprot.2007.199

[CIT0068] Zhan X, Qian B, Cao F, et al 2015 An *Arabidopsis* PWI and RRM motif-containing protein is critical for pre-mRNA splicing and ABA responses. Nature Communications 6, 8139. 10.1038/ncomms9139 PMC5514415 26404089

[CIT0069] Zhang Z, Zhang S, Zhang Y, et al 2011 *Arabidopsis* floral initiator SKB1 confers high salt tolerance by regulating transcription and pre-mRNA splicing through altering histone H4R3 and small nuclear ribonucleoprotein LSM4 methylation. The Plant Cell 23, 396–411. 21258002 10.1105/tpc.110.081356 PMC3051234

[CIT0070] Zhu JK 2002 Salt and drought stress signal transduction in plants. Annual Review of Plant Biology 53, 247–273. 10.1146/annurev.arplant.53.091401.143329 PMC3128348 12221975

[CIT0071] Zou M, Guan Y, Ren H, Zhang F, Chen F 2007 Characterization of alternative splicing products of bZIP transcription factors OsABI5. Biochemical and Biophysical Research Communications 360, 307–313. 17604002 10.1016/j.bbrc.2007.05.226

