# Supplementary material for: Arabidopsis exoribonuclease USB1 interacts with the PPR-domain protein SOAR1 to negatively regulate abscisic acid signaling
Source: J Exp Bot. 2020 Sep 24;71(19):5837–51. doi: 10.1093/jxb/eraa315 (PMC7541913; doi:10.1093/jxb/eraa315)
Supplement: eraa315_suppl_Supplementary_Material [file eraa315_suppl_supplementary_material.pdf]

Supplementary Data

Arabidopsis exoribonuclease USB1 interacts with the PPR-domain protein SOAR1 to negatively regulate abscisic acid signaling

Yu Ma*, Shang Zhang*,Chao Bi*, Chao Mei, Shang-Chuan Jiang, Xiao-Fang Wang, Zhi John Lu†, Da-Peng Zhang†

MOE Key Lab of Bioinformatics, Center for Plant Biology, School of Life Sciences, Tsinghua University, Beijing 100084, China

* These authors contributed equally to this work.

† To whom correspondence should be addressed. E-mail:zhangdp@tsinghua.edu.cn or zhilu@tsinghua.edu.cn

Supplementary Figure S1. Phylogenetic analysis of *Arabidopsis* USB1 protein homologues.
Supplementary Figure S2. Expression profile of the *USB1* gene and subcellular localization of USB1 protein.
Supplementary Figure S3. Early seedling growth of the *usb1* mutants under higher concentrations of ABA.
Supplementary Figure S4. Early seedling growth of the *usb1* mutants and *USB1*-overexpression lines, assayed by transferring germinating seeds to ABA-containing medium.
Supplementary Figure S5. Phenotypic analysis of the complementation lines of the *usb1*mutants: expression of *USB1* rescues the ABA-hypersensitive phenotypes of the *usb1*mutants.
Supplementary Figure S6. Early seedling growth of differentgenotypes under salt stress.
Supplementary Figure S7. Early seedling growth of differentgenotypes under the D-mannitol-induced osmotic stress.
Supplementary Figure S8. USB1functionally interacts with SOAR1 in ABA-induced early seedling growth inhibition.
Supplementary Figure S9. Quantification of alternative splicing events.
Supplementary Figure S10. Gene ontology analysis of co-regulated genes by USB1 and SOAR1 under Mock and ABA treatment conditions.
Supplementary Figure S11. The intron retention eventsof *HAB1*in the different genotypes.
Supplementary Figure S12. Diagrams of the intron retention events of the *CIPK3*, *MYB9* and*AT1G14170* in the different genotypes under Mock and ABA treatment conditions.
Supplementary Figure S13. Loss-of-function of *USB1* does not affect *ABI5* expression.
Supplementary Figure S14. Phenotypic observations of *usb1* mutants and *USB1*-overexpression lines during the life cycle of these plants.
Supplementary Table S1. Primers used in this study.
Supplementary Table S2. RNA-seq data: Function of genes co-regulated by USB1 and SOAR1.

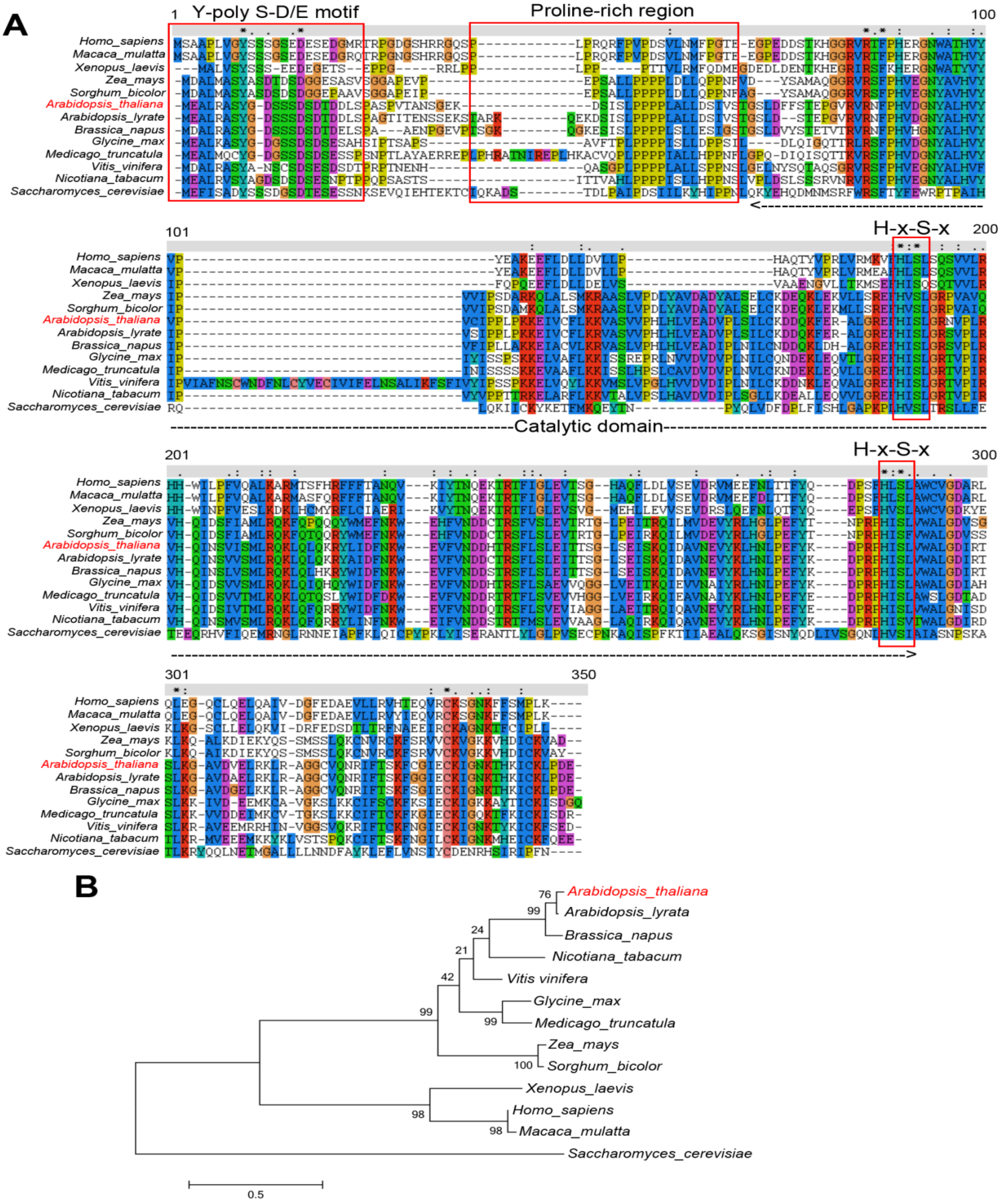

Supplementary Figure S1. Phylogenetic analysis of *Arabidopsis* USB1 protein homologues. (A) Aligment (using ClastalW software) of protein sequences of *Arabidopsis thaliana* (NP_568753.1), *Arabidopsis lyrata* (XP_002865845.2), *Brassica napus* (XP_013699104.1) ,*Nicotiana tabacum* (XP_016445810.1), *Vitis vinifera* (RVX01802.1), *Glycine max* (XP_003516818.1), *Medicago truncatula* (XP_024630396.1), *Zea mays* (ALI87299.1), *Sorghum bicolor* (XP_002452828.2), *Homo sapiens* (NP_078874.2), *Saccharomyces cerevisiae* (KZV09377.1), *Macaca mulatta* (XP_014981765.1), and *Xenopus laevis* (NP_001079479.1). Conserved amino acid residues are indicated by different colors. The symbol “*” indicates positions which have a single, fully

conserved amino acid residue, “:” the amino acids residue with strongly similar properties, and “.” the amino acids residue with weakly similar properties. Note that USB1 proteins contain two regions of sequence conservation near the N-terminus (with a conserved Y-poly S-(D/E) motif) and in the center of the N-terminal region (the proline-rich region), and exhibit a typical 2H phosphodiesterase folded with an active site containing two H-x-S-x motifs.The conserved domains are characterized as described previously (Zuo and Deutscher, 2001, Nucleic Acids Research,1; 29 (5): 1017–1026, https://doi.org/10.1093/nar/29.5.1017). (B) Ancestral states were analyzed using the Maximum Likelihood (ML) method and JTT matrix-based model (Jones DT, Taylor WR, Thornton JM.1992. The rapid generation of mutation data matrices from protein sequences. *Computer Applications in the Biosciences* 8, 275-282). Evolutionary analyses were conducted in MEGA 5.2 with a Bootstrap of 1000.

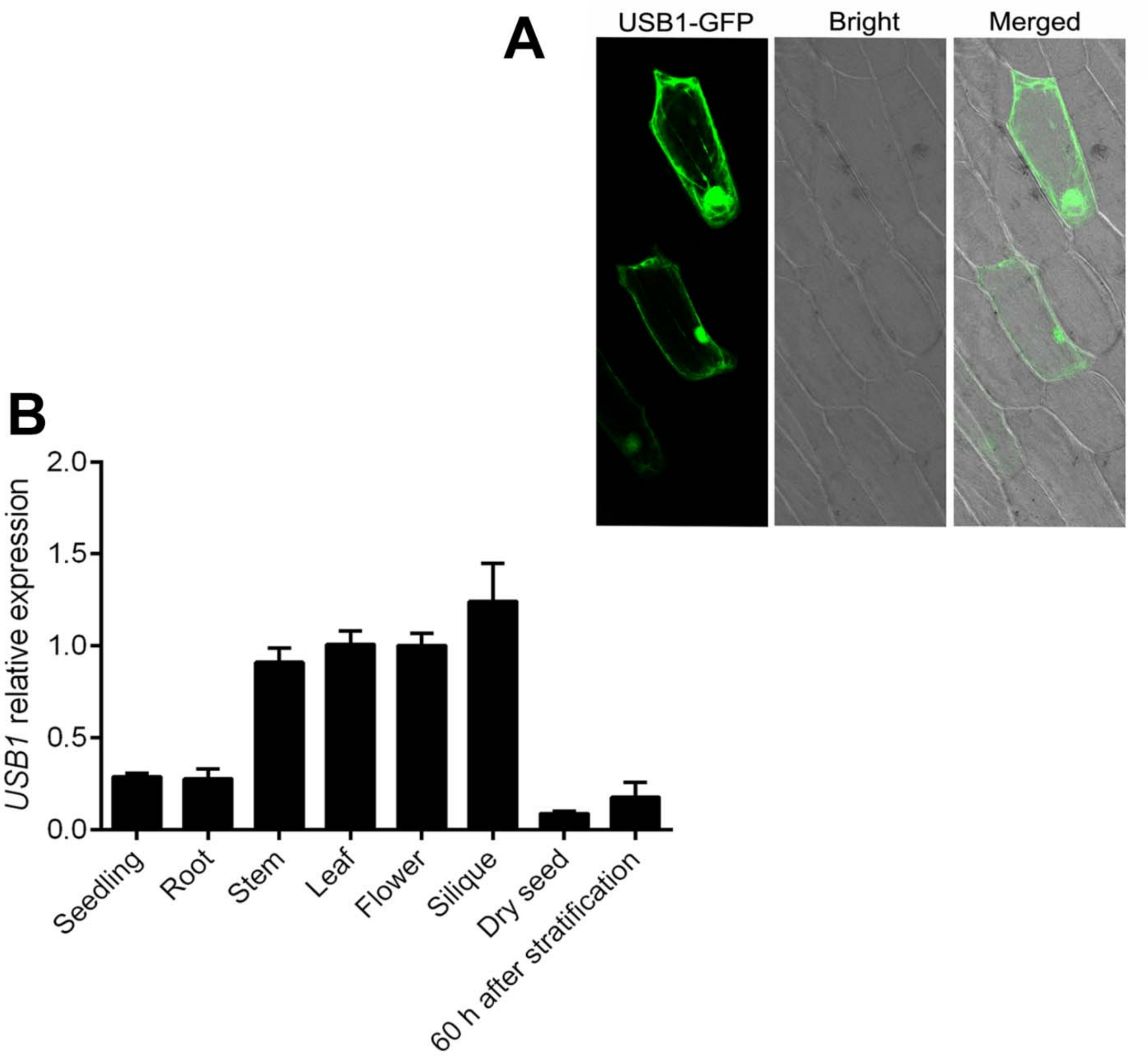

Supplementary Figure S2. Expression profile of the *USB1* gene and subcellular localization of USB1 protein. (A) USB1 is likely to localize to both the nucleus and cytoplasm. USB1 was fused withGFP (USB1-GFP) to form the USB1-GFP fusion protein, and transiently expressed in the onion epidermis cells. Bright, bright-field; Merged, merged images of the USB1-GFP signal with the bright field. The experiments were repeated five times with similar results. (B) Relative expression levels of *USB1* in different tissues/organs determined by quantitative real-time PCR. Each value is the mean ± SE of three independent biological determinations.

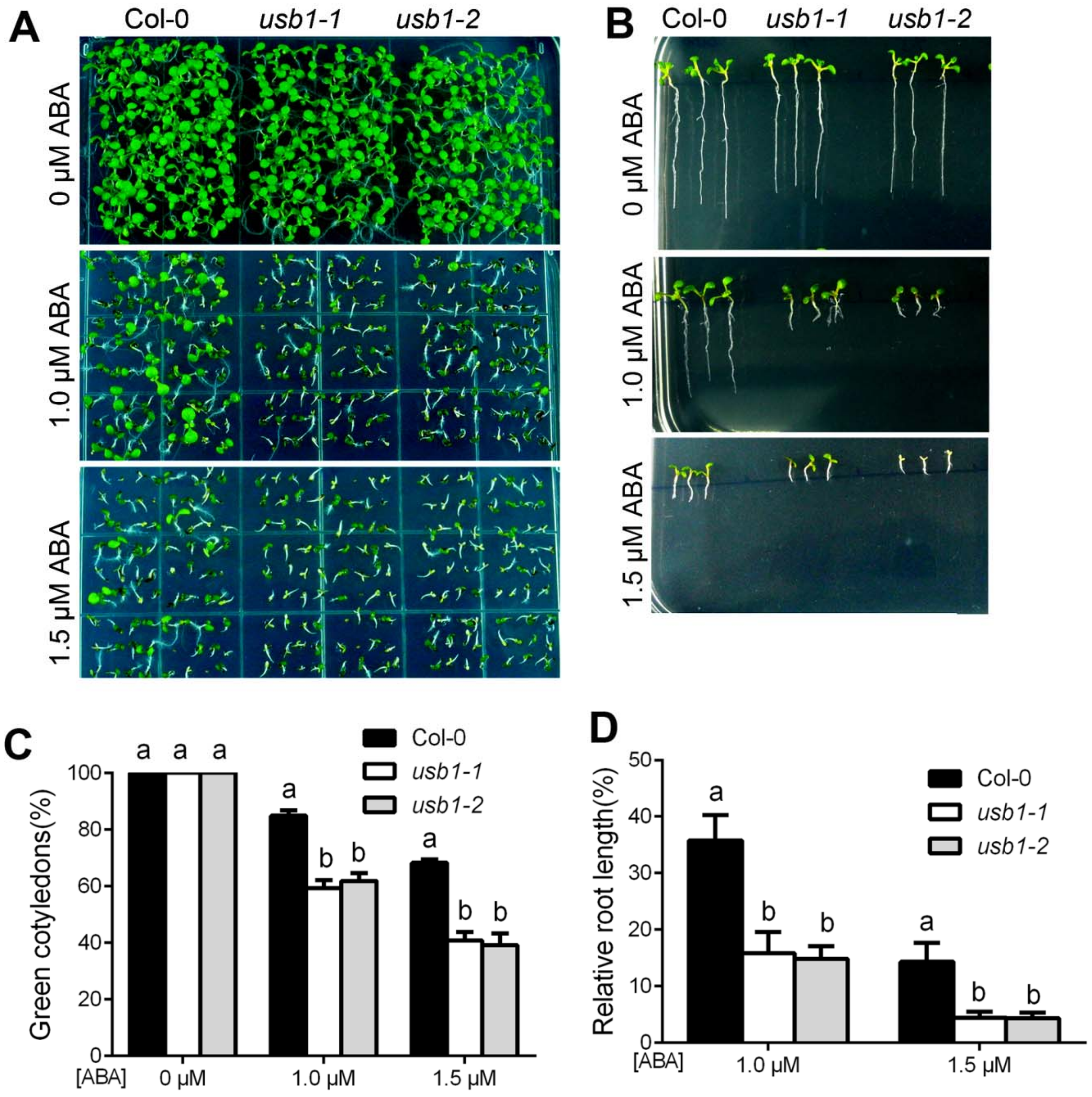

Supplementary Figure S3. Early seedling growth of the *usb1* mutants under higher concentrations of ABA. (A, B) Seedling growth of wild-type Col-0, *usb1-1* and *usb1-2* mutants in the (±)ABA-free MS medium (0 μM) and MS medium containing 1.0 or1.5 μM (±)ABA. Seeds were directly sown in the mediumand thegrowth was investigated 10 d after stratification at 4 °C for 3 d. (C) Statistical analysis of the rates of green cotyledons of different genotypes described in (A, B). (D) Statistical analysis of the relative root length of the different genotypes shown in (A, B). Relative values of the root length of each genotype grown on the ABA-containing MS medium were normalized relative to the value of the corresponding genotype at 0 μM ABA, which was taken as 100%.Each value is the mean ± SE of five biological determinations and different letters indicate significant differences at $P < 0.05$ (Duncan's multiple range test) when comparing values within the same ABA concentration.

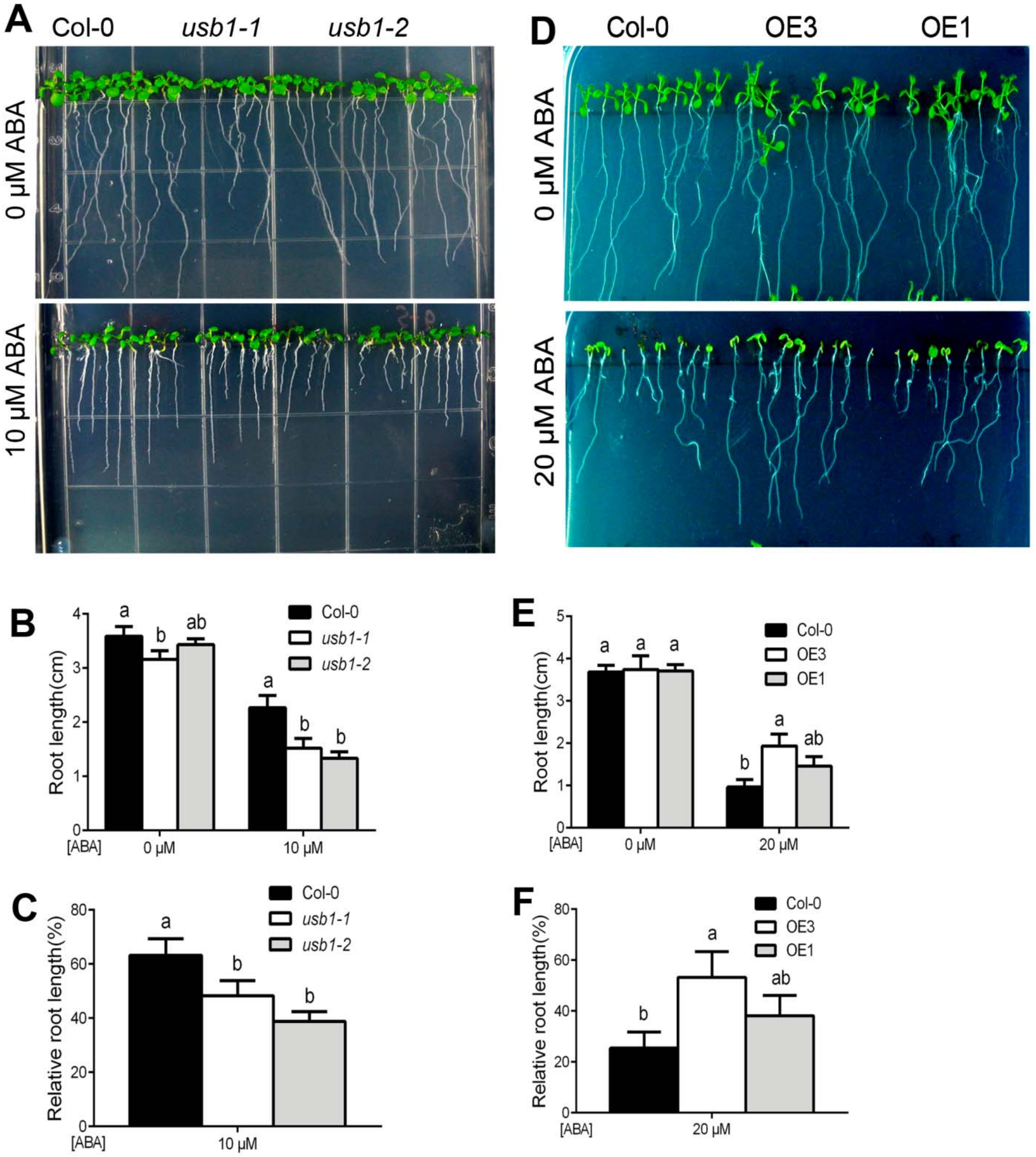

Supplementary Figure S4. Early seedling growth of the *usb1* mutants and *USB1*-overexpression lines, assayed by transferring germinating seeds to ABA-containing medium. Germinating seeds/young seedlings of the wild-type Col-0, *usb1-1* and*usb1-2* mutants (A-C) or Col-0, *USB1*-overexpression lines OE1 and OE3 (D-F) were transferred, 60 h after stratification(at 4 °C for 3 d), from ABA-free MS medium to the MS medium supplemented with (±)ABA at 0 (A-F), 10 (A-C), or 20 µM (D-F), and the growth was investigated 10 d after the seedling transfer.Statistical analysis data of the root length and relative root length of different genotypes described in (A) and (D) are shown in (B, C) and (E, F), respectively. Relative values of root length of each genotype grown on the

ABA-containing MS medium were normalized relative to the value of the corresponding genotype at 0 μM ABA, which was taken as 100%. Each value is the mean ± SE of five biological determinations and different letters indicate significant differences at $P < 0.05$ (Duncan's multiple range test) when comparing values within the same ABA concentration.

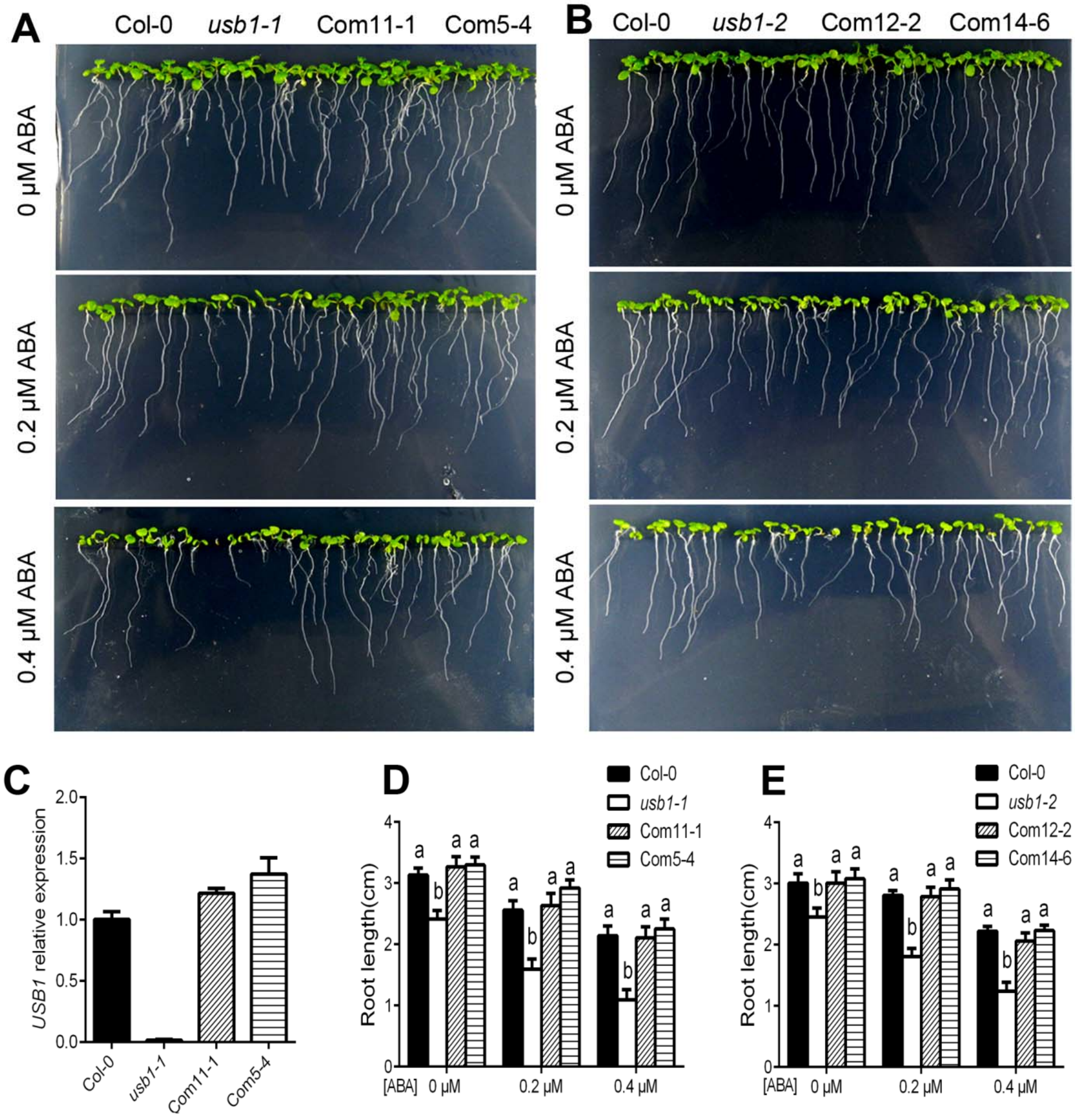

Supplementary Figure S5. Phenotypic analysis of the complementation lines of the *usb1* mutants: expression of *USB1* rescues the ABA-hypersensitive phenotypes of the *usb1* mutants. (A, B) Early seedling growth of Col-0, *usb1-1* and *usb1-1* complementation lines Com11-1 and Com5-4 (A), and Col-0, *usb1-2* and *usb1-2* complementation lines Com12-2 and Com14-6 (B), respectively. Seeds were directly sown in the (±)ABA-free (0 μM) or (±)ABA-containing medium and the growth was investigated 10 d after stratification at 4 °C for 3 d. (C) Quantitative real-time PCR analysis of the *USB1* expression level in wild-typeCol-0, *usb1-1* and complementation lines Com11-1 and Com5-4 seedlings. *ACTIN2/8* genes were used as internal controls, and the expression level of *USB1* in Col-0 was standardized as one unit. (D, E) Statistical analysis of the root length of different genotypes described in (A) and (B), respectively. Each value is the mean ± SE of five biological determinations and different letters indicate significant differences at $P < 0.05$ (Duncan's multiple range test) when comparing values within the same ABA concentration.

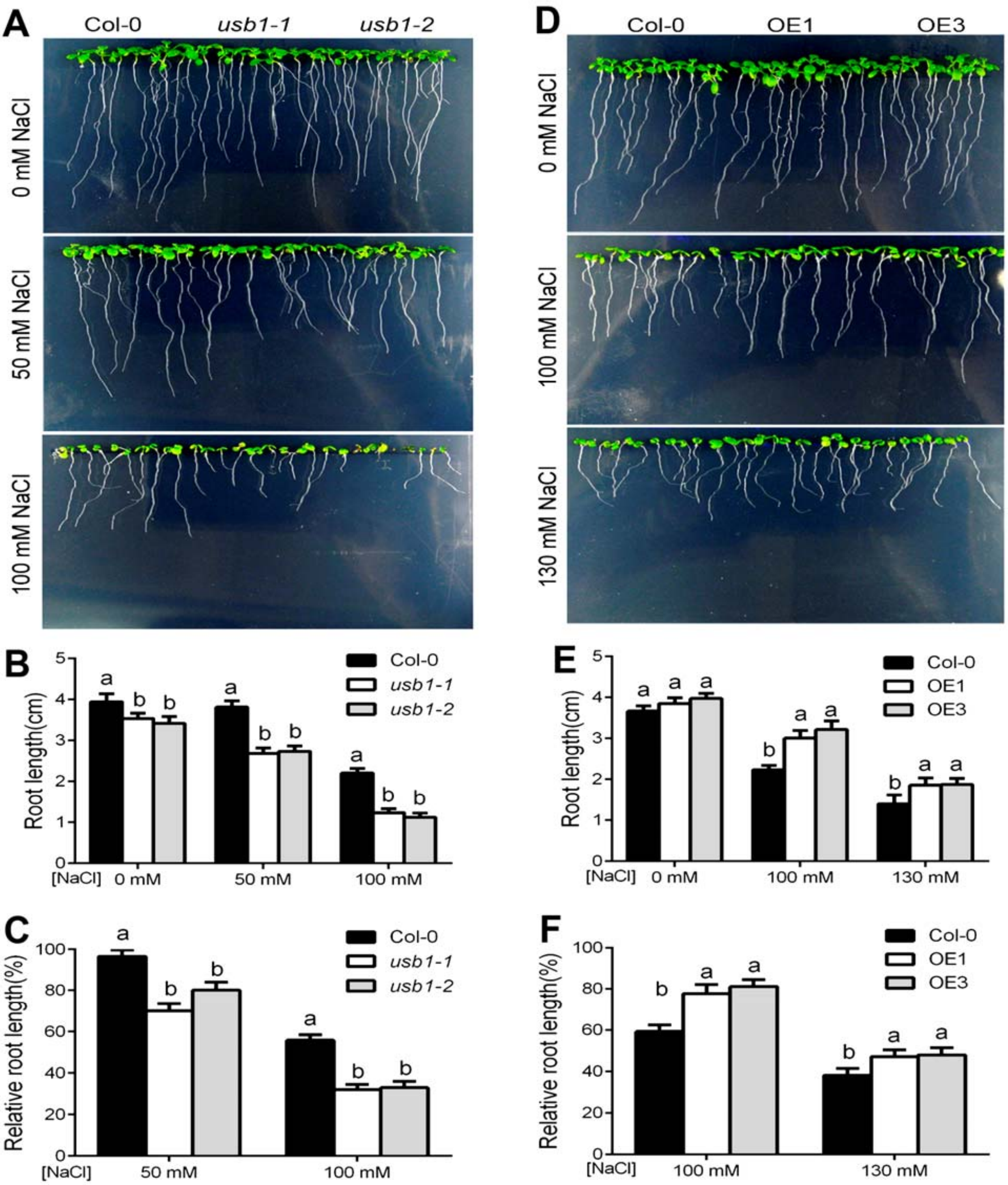

Supplementary Figure S6. Early seedling growth of different genotypes under salt stress. Seeds were directly planted in the NaCl-free MS medium (0 μM) or NaCl-containing (50, 100 mM in A-C, and 100, 130 mM in D-F) MS medium, and the seedling growth was investigated 10 d after stratification at 4 °C for 3 d. (A-C) Seedling growth of wild-type Col-0, *usb1-1*and*usb1-2* mutants under salt stress. Statistical analysis data of the absolute (B) and relative values (C) of root length of different genotypes described in (A) are shown. (D-F) Seedling growth of wild-type Col-0 and two *USB1*-overexpression lines (OE1, OE3) under salt stress. Statistical analysis data of root length (E) and relative root length (F) of different genotypes described in (D) are shown. In (C, F), relative values

of root length of each genotype grown on the NaCl-containing MS medium were normalized relative to the value of the corresponding genotype at 0 mM NaCl, which was taken as 100%.Each value is the mean ± SE of five biological determinations and different letters indicate significant differences at $P < 0.05$ (Duncan's multiple range test) when comparing values within the same NaCl concentration.

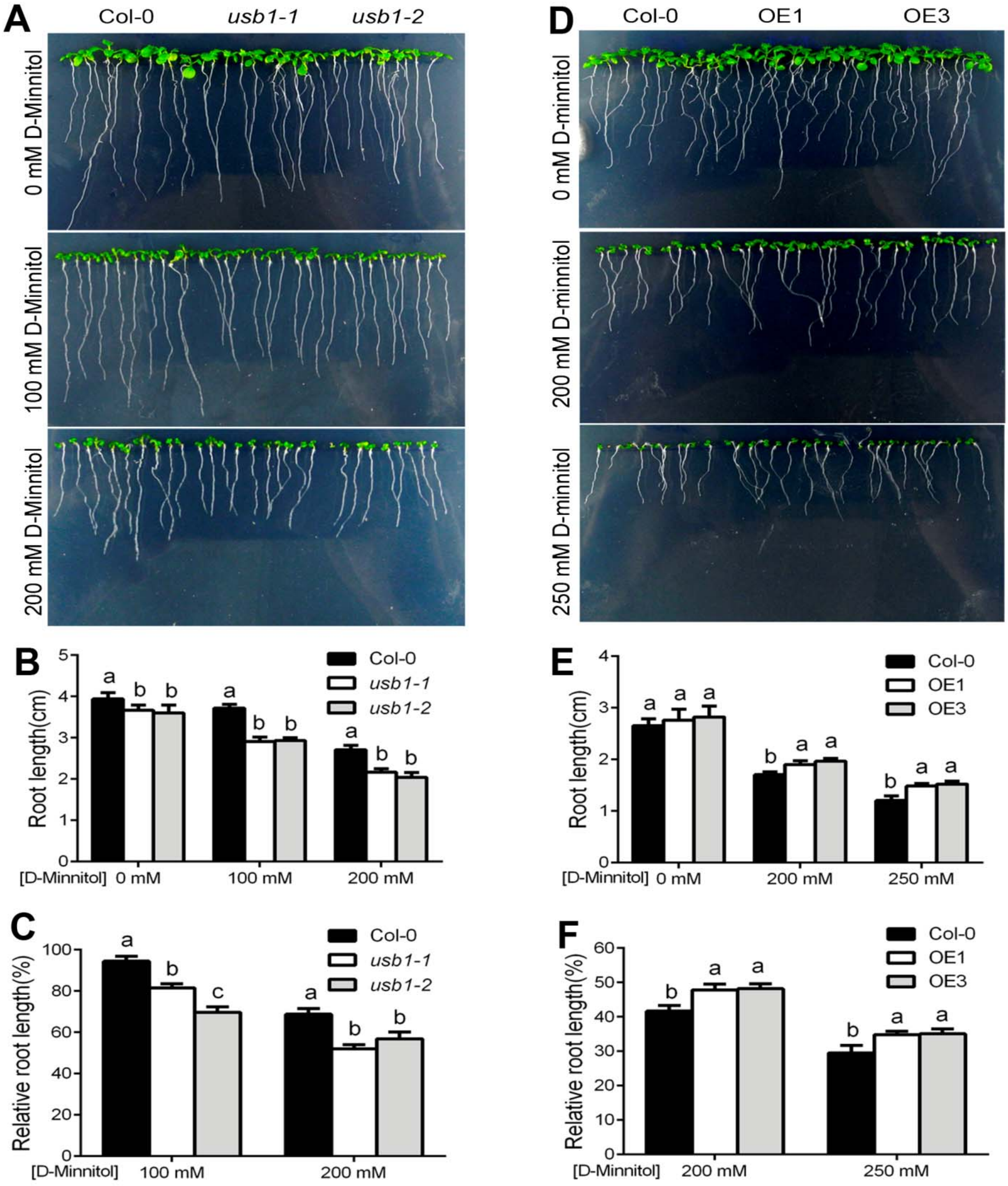

Supplementary Figure S7. Early seedling growth of different genotypes under the D-mannitol-induced osmotic stress. Seeds were directly planted in the D-mannitol-free MS medium (0 μM) or D-mannitol-containing (100, 200 mM in A-C, and 200, 250 mM in D-F) MS medium, and the seedling growth was investigated 10 d after stratification at 4 °C for 3 d. (A-C) Seedling growth of wild-type Col-0, *usb1-1* and *usb1-2* mutants under D-mannitol-induced osmotic stress. Statistical analysis data of the absolute (B) and relative values (C) of root length of different genotypes described in (A) are shown. (D-F) Seedling growth of wild-type Col-0 and two *USB1*-overexpression lines (OE1, OE3) under D-mannitol-induced osmotic stress. Statistical analysis data of root

length (E) and relative root length (F) of different genotypes described in (D) are shown. In (C, F), relative values of root length of each genotype grown on the D-mannitol-containing MS medium were normalized relative to the value of the corresponding genotype at 0 mM D-mannitol, which was taken as 100%. Each value is the mean ± SE of five biological determinations and different letters indicate significant differences at $P < 0.05$ (Duncan's multiple range test) when comparing values within the same D-mannitol concentration.

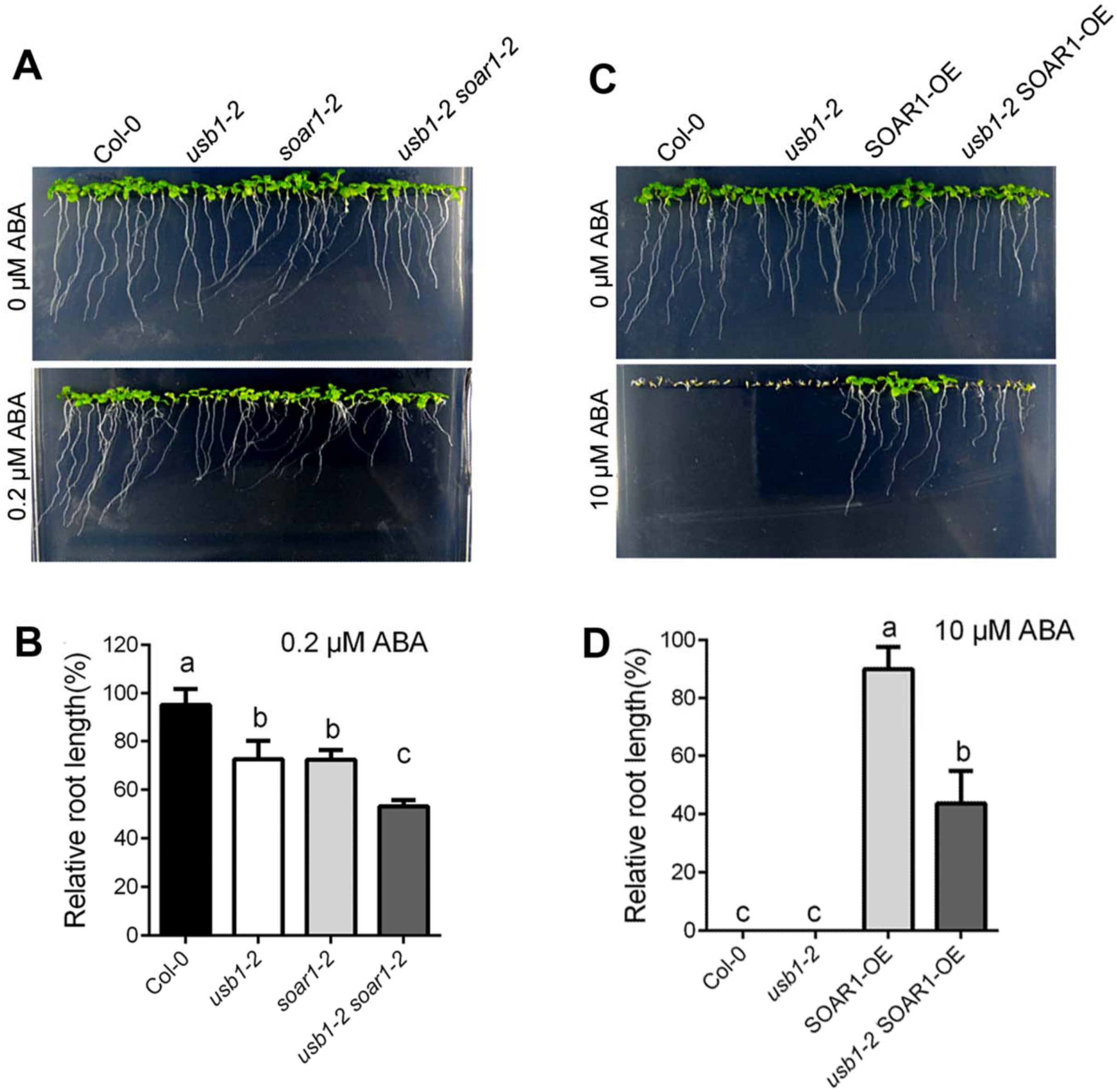

Supplementary Figure S8. USB1 functionally interacts with SOAR1 in ABA-induced early seedling growth inhibition. (A, B) Seedling growth of the wild-type Col-0, *usb1-2* and *soar1-2* single mutants and *usb1-2 soar1-2* double mutant in ABA-free (0 μM) and ABA-containing (0.2 μM) medium 10 d after stratification at 4 °C for 3 d. (C, D) Seedling growth of wild-type Col-0, *usb1-2*, *SOAR1*-overexpression line OE6 (SOAR1-OE), and SOAR1-OE under *usb1-2* background (*usb1-2* SOAR1-OE) in ABA-free (0 μM) and ABA-containing(10 μM) medium. The column figures show statistical analysis of the relative root length of the corresponding different genotypes, and the relative values of root length of each genotype grown on the ABA-containing MS medium were normalized relative to the value of the corresponding genotype at 0 μM ABA, which was taken as 100%. Each value is the mean ± SE of five biological determinations and different letters indicate significant differences at $P < 0.05$ (Duncan's multiple range test) when comparing values within the same ABA concentration.

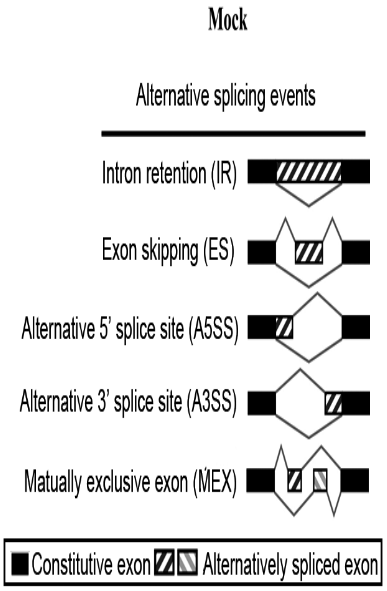

	Col-0_vs_*usb1-1*			Col-0_vs_*soar1-2*			Col-0_vs_*usb1-1 soar1-2*		
	No.events	Frequenc (%)	No.genes	No.events	Frequency (%)	No.genes	No.events	Frequency (%)	No.genes
IR	263	45.7	244	125	34.8	114	247	40	231
ES	210	36.5	181	113	31.5	103	199	32.1	176
A5SS	32	5.5	31	20	5.6	19	49	7.9	48
A3SS	64	11.1	63	87	24.2	86	108	17.4	104
MEX	7	1.2	7	14	3.9	12	16	2.6	14
Total	576	100	526	359	100	334	619	100	573

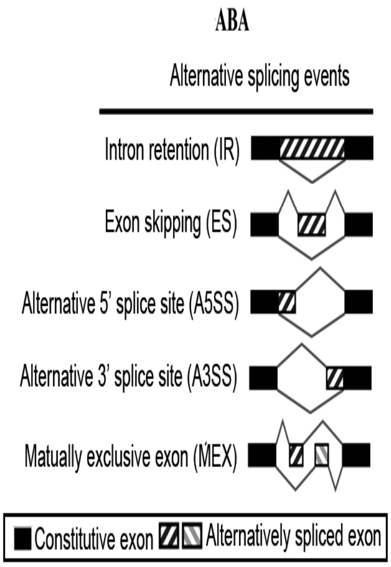

	Col-0_vs_*usb1-1*			Col-0_vs_*soar1-2*			Col-0_vs_*usb1-1 soar1-2*		
	No.events	Frequency (%)	No.genes	No.events	Frequency (%)	No.genes	No.events	Frequency (%)	No.genes
IR	213	45.7	197	119	33.7	109	253	39.8	237
ES	173	37.1	151	122	34.6	108	224	35.2	196
A5SS	25	5.4	24	21	5.9	21	44	6.9	43
A3SS	53	11.4	52	72	20.4	71	98	15.4	93
MEX	2	0.4	2	19	5.4	17	17	2.7	15
Total	466	100	426	353	100	326	636	100	584

Supplementary Figure S9. Quantification of alternative splicing events (IR, ES, A5SS, A3SS, MEX as indicated) identified in the *usb1-1* and *soar1-2* single mutants and *usb1-1 soar1-2* double mutant in comparison to the wild-type Col-0 under Mock (top) and ABA treatment (bottom) conditions.

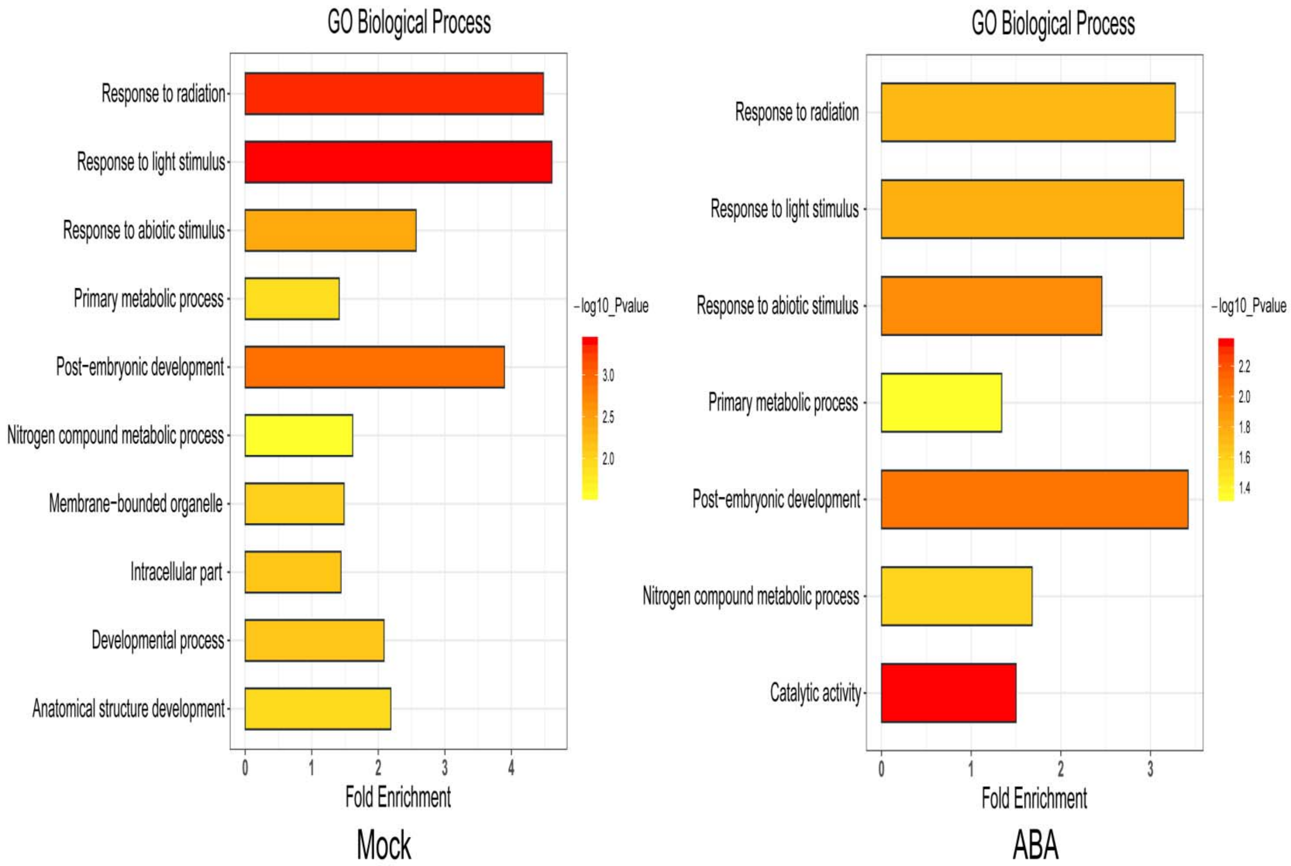

Supplementary Figure S10. Gene ontology(GO) analysis of co-regulated genes by USB1 and SOAR1 under Mock (left) and ABA treatment (right) conditions. Gene ontology classifications were at least 1.2-fold enriched and with a statistical significance of $P < 0.05$. Red color indicates the P-value of gene ontology terms (expressed as -log10 _Pvalue).

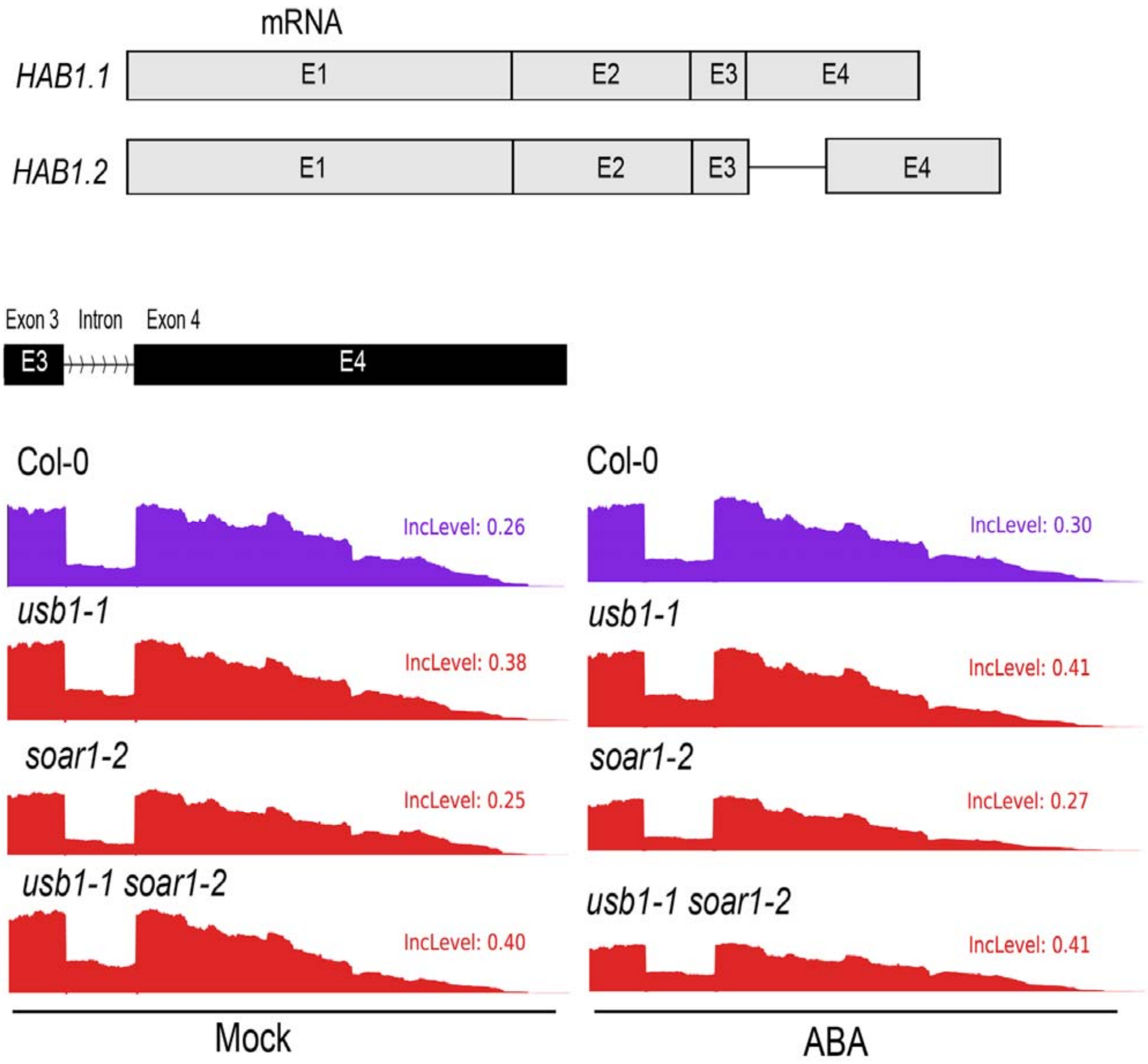

Supplementary Figure S11. The intron retention events of *HAB1* in the different genotypes. Top: diagrams of the *HAB1.1* and *HAB1.2* variants (figure drawn to scale). E1-E4 represent exon1-4. Bottom: the intron retention events, visualized by the Rmats2sashimiplot in the wild-type Col-0, *usb1-1* and *soar1-2* single mutants and *usb1-1 soar1-2* double mutant under Mock and ABA treatment conditions. A diagram indicating the structure of the corresponding domain in the *HAB1* pre-mRNA (Exon 3-Intron-Exon 4) for the Rmats2sashimiplot analysis, is shown. IncLevel, inclusion level (ψ), indicates the percentage of reads mapped to the intron inclusion transcripts among reads mapped to intron inclusion transcripts plus intron spliced transcripts. The difference in the mean ψ values between wide type and mutants exceeding 0.05 (|IncLevel Difference|>0.05 or $|\Delta\psi| = |\psi_{i1}-\psi_{i2}|>0.05$) and P value < 0.05 means significantly and differentially alternative splice events (Shen, S., Park, J.W., Lu, Z.X., Lin, L., Henry, M.D., Wu, Y.N., Zhou, Q., and Xing, Y. 2014. rMATS: robust and flexible detection of differential alternative splicing from replicate RNA-Seq data. *Proc Natl Acad Sci USA* 111: E5593-5601).

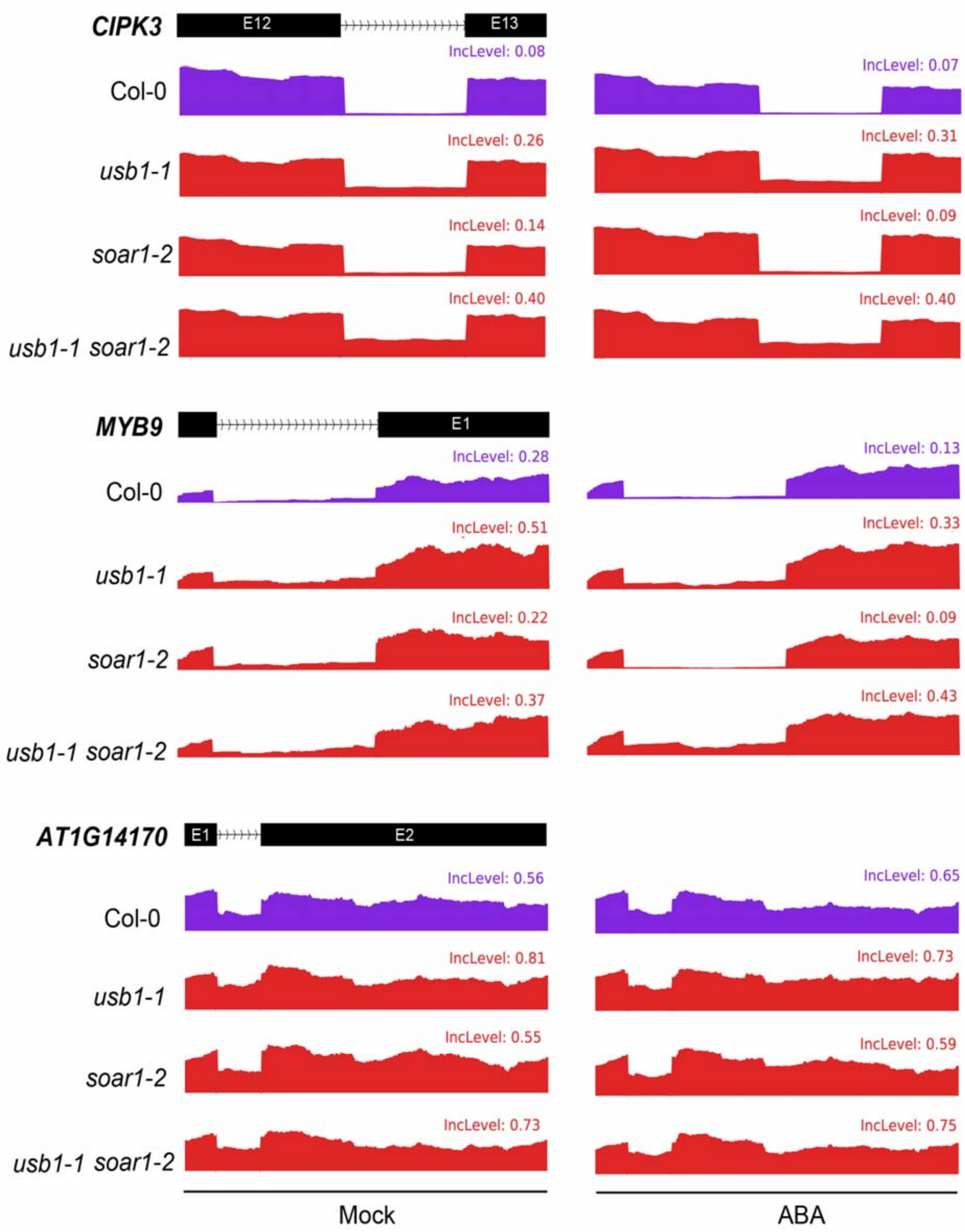

Supplementary Figure S12. Diagrams of the intron retention events of the *CIPK3*, *MYB9* and *AT1G14170* in the different genotypes (as indicated) under Mock (left panels, which have been shown earlier in the Fig. 5) and ABA treatment (right panels) conditions. The retained introns (dashed lines) with their relative positions in the representative gene model and the flanking exons (black bars), together with the captures of the corresponding read coverage tracks visualized by the Rmats2sashimiplot, are shown. E represents exon. IncLevel (Inclusion level: ψ)

indicates the percentage of reads mapped to the intron inclusion transcripts among reads mapped to intron inclusion transcripts plus intron spliced transcripts. The difference in the mean of ψ values between wide type and mutants exceeding 0.05 (|IncLevel Difference|> 0.05 or $|\Delta\psi| = |\psi_{i1} - \psi_{i2}| > 0.05$) and P value < 0.05 means significantly and differentially alternative splice events (Shen, S., Park, J.W., Lu, Z.X., Lin, L., Henry, M.D., Wu, Y.N., Zhou, Q., and Xing, Y. 2014. rMATS: robust and flexible detection of differential alternative splicing from replicate RNA-Seq data. *Proc Natl Acad Sci USA* 111: E5593-5601).

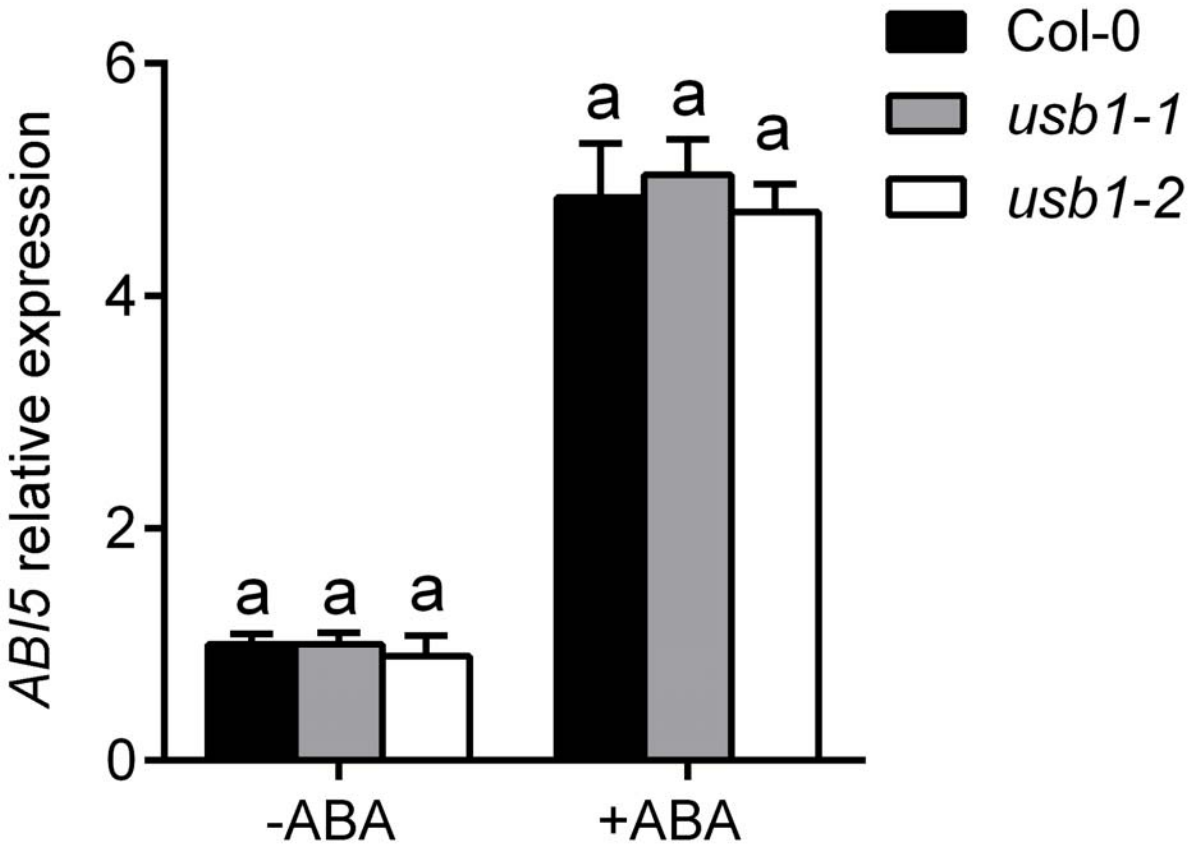

Supplementary Figure S13. Loss-of-function of *USB1* does not affect *ABI5* expression. Relative expression levels of *ABI5* in the *usb1-1* and *usb1-2* mutants treated by ABA-free (-ABA) or 10 μM-ABA treatment, were determined by quantitative real-time PCR. Each value is the mean ± SE of three independent biological determinations and different letters indicate significant differences at $P < 0.05$ (Duncan's multiple range test) when comparing values within the same ABA concentration.

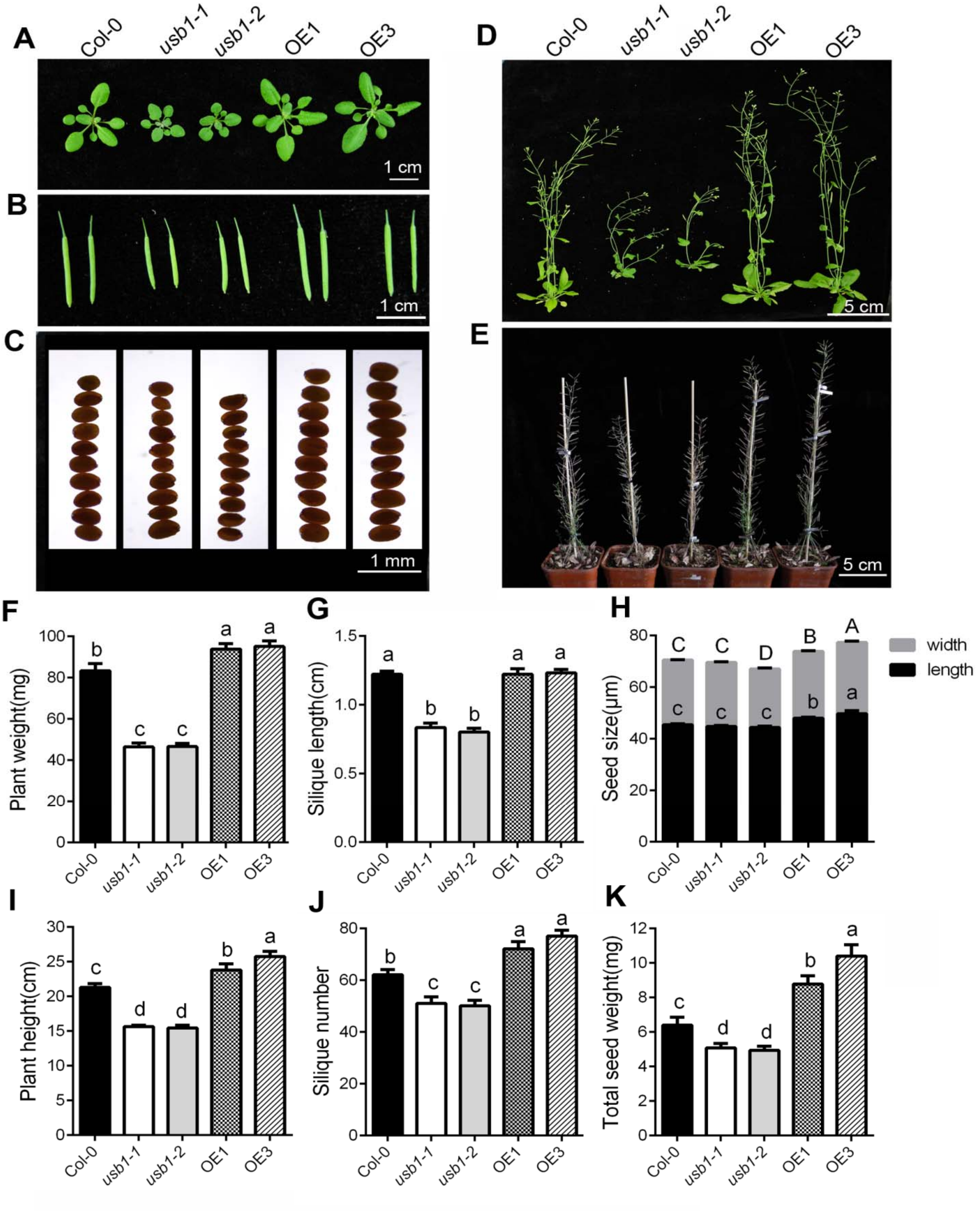

Supplementary Figure S14. Phenotypic observations of *usb1* mutants and *USB1*-overexpression lines during the life cycle of these plants.(A-E) Phenotypes of wild-type Col-0, *usb1-1* and*usb1-2* mutants, and *USB1*-overexpression lines OE1 and OE3 plants were investigated. Four-week-old seedlings (A), siliques (B), seeds (C), 6-week-old plants (D), 9-week-old plants (E). (F-K) Statistical data of the plant weight (F) described in (A), siliques length (G) described in (B), seed size (H) described in (C), plant height (I) described in (D), silique

number (G) and total seed weight described in (E) are shown. Each value is the mean ± SE of five biological determinations and different letters indicate significant differences at $P < 0.05$ (Duncan's multiple range test).

Supplementary Table S1. Primers Used in This Study.

1. Primers for identification the mutants.LP, left primer; RP, right primer

LBb1	5’-ATTTTGCCGATTTCGGAAC-3’
usb1-1 (SAIL_717_G03)_LP	5’-ATCTCCAAAATCCCCATCATC-3’
usb1-1 (SAIL_717_G03)_RP	5’-GATACACTTCATCAGGCCTGC-3’
LB4	5’-CGTGTGCCAGGTGCCCACGGAATAGT-3’
soar1-2(FLAG_546D07)_LP	5’-GTGAACCAACTCAACACTCGG-3’
soar1-2(FLAG_546D07)_RP	5’-TCACCGCAATGTATCTACCATC-3’

2. Primers for generating *usb1-2* mutant. F: forward primer; R: reverse primer

usb1-2-F	5’-attgATACCTCCACTGCCAAAGA-3’
usb1-2-R	5’-aaacTCTTTGGCAGTGGAGGTAT-3’

3. Primers for generating the transgenic over-expression lines and complementation lines of the *usb1-1* and *usb1-2* mutants.

USB1-GFP-F	5’-CCttaattaaATGGAAGCATTGAGAGC-3’
USB1-GFP-R	5’-AggcgcgccTTCATCTGGGAGTTTAC-3’

*The underlines represent the enzyme restriction sites.

4. Primers for *in vitro* pull-down assay.

USB1-GST-F	5’-CCGgaattcATGGAAGCATTGAGAGC-3’
USB1-GST-R	5’-CCGctcgagTTATTCATCTGGGAG-3’
SOAR1-His-F	5’-ATAAGAATgcggccgcATGAACTCTCTGTTCA CCGC-3’
SOAR1-His-R	5’-ACGCgtcgacTTAGTGATGATGATGATGATGCTC AAAATCCCCTGCATCTC-3’

*The underlines represent the enzyme restriction sites.

5. Primers for luciferase complementation imaging assays (LCI).

USB1-NLuc-F	5’-CGggatccATGGAAGCATTGAGAGC-3’
USB1-NLuc-R	5’-ACGCgtcgacTTCATCTGGGAGTTTAC-3’
CLuc-SOAR1-F	5’-GGggtaccATGAACTCTCTGTTC-3’
CLuc-SOAR1-R	5’-ACGCgtcgacTCACTCAAAATCCCCTGC-3’
SOAR1-NLuc-F	5’-CGggatccATGAACTCTCTGTTC-3’
SOAR1-NLuc-R	5’-ACGCgtcgacCTCAAAATCCCCTGCA-3’

CLuc-USB1-F	5’-GGggtaccATGGAAGCATTGAGAGC-3’
CLuc-USB1-R	5’-ACGCgtcgacTTATTCATCTGGGAGTT-3’

*The underlines represent the enzyme restriction sites.

6. Primers for bimolecular imaging of fluorescent complementation assay (BiFC).

USB1-NYFP (CYFP)-F	5’-TTggcgcgccATGGAAGCATTGAGAGC-3’	BiFC in *A. thaliana*
USB1-NYFP (CYFP)-R	5’-ACGCgtcgacTTCATCTGGGAGTTTAC-3’	
SOAR1-CYFP (NYFP)-F	5’-GCtctagaATGAACTCTCTGTTCACC-3’	
SOAR1-CYFP (NYFP)-R	5’-GGggtaccCTCAAAATCCCCTGCATC-3’	
USB1-NYFP-F	5’-cgacgacaagaccgtcATGGAAGCATTGAGAGC-3’	BiFC in *N. benthamiana*
USB1-NYFP-R	5’-gaggagaagagccgtcgTTCATCTGGGAGTTTAC-3’	
SOAR1-CYFP-F	5’-cgacgacaagaccgtcATGAACTCTCTGTTCAC-3’	
SOAR1-CYFP-R	5’-gaggagaagagccgtcgCTCAAAATCCCCTGCAT-3’	

*The underlines represent the enzyme restriction sites.

7. Primers for subcellular localization of the USB1.

USB1-GFP-F	5’-CCGgaattcATGGAAGCATTGAGAGC-3’
USB1-GFP-R	5’-ACGCgtcgactgTTCATCTGGGAGTTTAC-3’
SOAR1-RFP-F	5’-CCttaattaaATGAACTCTCTGTTC-3’
SOAR1-RFP-R	5’-AggcgcgccACTCAAAATCCCCTG-3’
FBI1-RFP-F	5’-CCttaattaaATGTCGAATAATCAAGCTTT-3’
FBI1-RFP-R	5’-AggcgcgccATAGTCTTCTCATCGCATGGG-3’

*The underlines represent the enzyme restriction sites.

8. Primers for qRT-PCR.

USB1-qPCR-F	5’-AAGCATTGAGAGCGTCCTACG-3’
USB1-qPCR-R	5’-GTTTCTTACACGAACTCCAGGC-3’
SOAR1-qPCR-F	5’-TACGGAACTTAGGTTGCTTGAG-3’
SOAR1-qPCR-R	5’-AACAACAGTCGGCTTCACATTC-3’
ACTIN2/8-qPCR-F	5’-GGTAACATTGTGCTCAGTGGTGG-3’
ACTIN2/8-qPCR-R	5’-AACGACCTTAATCTTCATGCTGC-3’

9. Primers for splicing efficiency assay. UF: unspliced forward primer; UR: unspliced reverse primer; SF: splicedforward primer; SR: spliced reverse primer.

HAB1-UF	5’-GCACATCACACAAACGTGGCTTGATAATG-3’
HAB1-SF	5’-CATGTCTAGGTCCATCGGTGACAGATATC-3’
HAB1-UR/SR	5’-CTTGGTTGTTCATTACATCCCAAAGACCG-3’
CIPK3-UF	5’-GTTAGTGAAAACTCTGTAACGGAAATGAA-3’
CIPK3-SF	5’-AAGAAGAACTACAAGATGAGGCTTGAGAAT-3’
CIPK3-UR/SR	5’-ACTTGGAATATCTCTGTCGCTACAT-3’

AT1G14170-UF	5’-GTGAGATACACTCACAATGATTAGAGA-3’
AT1G14170-SF	5’-ATGAGCTTCTTCTGATAATTGGAGAGC-3’
AT1G14170-UR/SR	5’-CATTGCACCAGGTTGATGCATGGAAGA-3’
MYB9-UF	5’-AGTGTGTTTGATTTATACATTATCTTTA-3’
MYB9-SF	5’-GGAGATCTTCAACAGATGGGGCGATCACCAT-3’
MYB9-UR/SR	5’-GGCTTGCTTTGGAAGAGCTCTCCA-3’

Supplementary Table S2. RNA-seq data: Function of genes co-regulated by USB1 and SOAR1

1. Mock

Function of genes	Number of genes	P value	Locus	Description
Response to abiotic stimulus	11	0.0039	AT4G38240	Complex glycanless, CGL1
			AT5G37370	PRP38 family protein,ATSRL1
			AT1G77080	MADS affecting flowering 1, MAF1
			AT2G41430	Early responsive to dehydration 15, ERD15
			AT2G47860	Phototropic-responsive NPH3 family protein, SETH6
			AT2G26980	CBL-interacting protein kinase 3, CIPK3
			AT5G48150	Phytochrome a signal transduction 1, PAT1
			AT1G69530	Expansin A1,EXPA1
			AT3G47340	Glutamine-dependent asparagine synthase 1, ASN1
			AT3G07650	CONSTANS-like 9, COL9
			AT1G01060	Late elongated hypocotyl, LHY1, LHY1
Response to light stimulus	8	0.00039	AT5G48150	Phytochrome a signal transduction 1, PAT1
			AT2G41430	Early responsive to dehydration 15, ERD15
			AT2G47860	Phototropic-responsive NPH3 family protein, SETH6
			AT1G01060	Late elongated hypocotyl, LHY1, LHY1
			AT1G77080	MADS affecting flowering 1, MAF1
			AT1G69530	Expansin A1,EXPA1
			AT3G47340	Glutamine-dependent asparagine synthase 1, ASN1
			AT3G07650	CONSTANS-like 9, COL9
Response to radiation	8	0.00047	AT5G48150	Phytochrome a signal transduction 1, PAT1
			AT2G41430	Early responsive to dehydration 15, ERD15
			AT2G47860	Phototropic-responsive NPH3 family protein, SETH6
			AT1G01060	Late elongated hypocotyl, LHY1, LHY1
			AT1G77080	MADS affecting flowering 1, MAF1
			AT1G69530	Expansin A1,EXPA1
			AT3G47340	Glutamine-dependent asparagine synthase 1, ASN1

			AT3G07650	CONSTANS-like 9, COL9
Post-embryonic development	8	0.0012	AT1G19080	GINS complex protein, TTN10
			AT4G23250	Cysteine-rich receptor-like protein kinase 17, CRK17
			AT1G77080	MADS affecting flowering 1, MAF1
			AT1G06220	Maternal effect embryo arrest 5, MEE5
			AT1G55350	Defective kernel, DEK1
			AT1G01060	Late elongated hypocotyl, LHY1, LHY1
			AT5G48150	Phytochrome a signal transduction 1, PAT1
			AT3G07650	CONSTANS-like 9, COL9
Developmental process	14	0.0073	AT1G19080	GINS complex protein, TTN10
			AT4G23250	Cysteine-rich receptor-like protein kinase 17, CRK17
			AT1G77080	MADS affecting flowering 1, MAF1
			AT1G06220	Maternal effect embryo arrest 5, MEE5
			AT1G55350	Defective kernel, DEK1
			AT1G01060	Late elongated hypocotyl, LHY1, LHY1
			AT5G48150	Phytochrome a signal transduction 1, PAT1
			AT3G07650	CONSTANS-like 9, COL9
			AT4G35770	Senescence associated gene 1, SEN1
			AT5G24630	Brassinosteroid-insensitive 4, BIN4
			AT1G05230	Homeodomain GLABROUS 2, HDG2
			AT2G02450	NAC domain containing protein 35, LOV1
			AT4G20380	Lesion simulating disease 1, LSD1
			AT1G69530	Expansin A1,EXPA1
Nitrogen compound metabolic process	18	0.028	AT5G37370	PRP38 family protein,ATSRL1
			AT1G16610	Arginine/Serine-rich, SR45
			AT1G19080	GINS complex protein, TTN10
			AT1G30810	Jumonji domain-containing protein 18, JMJ18
			AT1G54385	ARM repeat superfamily protein
			AT3G17040	High-chlorophyll fluorescent 107, HCF107
			AT5G17300	REVEILLE 1, RVE1
			AT5G24630	Brassinosteroid-insensitive 4, BIN4
			AT1G05230	Homeodomain GLABROUS 2, HDG2
			AT3G46600	GRAS family transcription factor
			AT4G29930	Basic helix-loop-helix (bHLH) DNA-binding superfamily protein
			AT4G20380	Lesion simulating disease 1, LSD1
			AT3G07650	CONSTANS-like 9, COL9

			AT1G77080	MADS affecting flowering 1, MAF1
			AT4G34570	Thymidylate synthase 2, THY-2
			AT1G01060	Late elongated hypocotyl, LHY1, LHY1
			AT3G47340	Glutamine-dependent asparagine synthase 1, ASN1
			AT1G13450	Homeodomain-like superfamily protein, GT-1
Intracellular part	39	0.0074	AT1G16010	Magnesium transporter 2, MGT2
			AT3G60250	Casein kinase II beta chain 3, CKB3
			AT3G45050	Transmembrane protein
			AT5G19950	Tudor domain protein, DUF1767
			AT1G79500	Aldolase-type TIM barrel family protein, ATKDSA1
			AT5G38290	Peptidyl-tRNA hydrolase family protein
			AT2G41540	GPDHC1
			AT1G58180	Beta carbonic anhydrase 6, BCA6
			AT1G21350	Thioredoxin superfamily protein
			AT3G27110	Peptidase family M48 family protein
			AT4G03200	Catalytics
			AT1G43620	UDP-Glycosyltransferase superfamily protein, UGT80B1
			AT4G33625	Vacuole protein
			AT5G08535	D111/G-patch domain-containing protein
			AT4G29930	Basic helix-loop-helix (bHLH) DNA-binding superfamily protein
			AT5G53180	Polypyrimidine tract-binding protein 2, PBT2
			AT3G26690	Nudix hydrolase homolog 13, NUDX13
			AT4G00440	GPI-anchored adhesin-like protein, putative DUF3741/TON1 Recruiting motif 15, TRM15
			AT4G35785	RNA-binding (RRM/RBD/RNP motifs) family protein
			AT5G65685	UDP-Glycosyltransferase superfamily protein
			AT5G48150	Phytochrome a signal transduction 1, PAT1
			AT1G06220	Maternal effect embryo arrest 5, MEE5
			AT1G77080	MADS affecting flowering 1, MAF1
			AT1G19080	GINS complex protein, TTN10
			AT1G30810	Jumonji domain-containing protein 18, JMJ18
			AT2G26980	CBL-interacting protein kinase 3, CIPK3

			AT1G16610	Arginine/Serine-rich, SR45
			AT5G24630	Brassinosteroid-insensitive 4, BIN4
			AT2G41430	Early responsive to dehydration 15, ERD15
			AT1G13450	Homeodomain-like superfamily protein, GT-1
			AT5G10490	MSCS-like 2, MSL2
			AT4G34265	Hypothetical protein
			AT2G36680	Modifier of rudimentary (Mod(r)) protein
			AT5G56500	TCP-1/cpn60 chaperonin family protein
			AT3G17040	High-chlorophyll fluorescent 107, HCF107
			AT3G07650	CONSTANS-like 9, COL9
			AT4G38240	Complexglycanless, CGL1
			AT1G05230	Homeodomain GLABROUS 2, HDG2
			AT4G00400	Glycerol-3-phosphate acyltransferase 8, GPAT8
Anatomical structure development	11	0.012	AT1G19080	GINS complex protein, TTN10
			AT4G23250	Cysteine-rich receptor-like protein kinase 17, CRK17
			AT1G77080	MADS affecting flowering 1, MAF1
			AT1G06220	Maternal effect embryo arrest 5, MEE5
			AT1G55350	Defective kernel, DEK1
			AT1G01060	Late elongated hypocotyl, LHY1, LHY1
			AT5G24630	Brassinosteroid-insensitive 4, BIN4
			AT1G05230	Homeodomain GLABROUS 2, HDG2
			AT4G20380	Lesion simulating disease 1, LSD1
			AT1G69530	Expansin A1, EXPA1
			AT3G07650	CONSTANS-like 9, COL9
Membrane-bounded organelle	33	0.0093	AT1G16010	Magnesium transporter 2, MGT2
			AT2G36680	Modifier of rudimentary (Mod(r)) protein
			AT3G17040	High-chlorophyll fluorescent 107, HCF107
			AT5G56500	TCP-1/cpn60 chaperonin family protein
			AT3G45050	Transmembrane protein
			AT5G19950	Tudor domain protein, DUF1767
			AT5G38290	Peptidyl-tRNA hydrolase family protein
			AT1G58180	Beta carbonic anhydrase 6, BCA6
			AT1G21350	Thioredoxin superfamily protein
			AT3G27110	Peptidase family M48 family protein
			AT4G03200	Catalytics

			AT1G43620	UDP-Glycosyltransferase superfamily protein, UGT80B1
			AT4G33625	Vacuole protein
			AT5G08535	D111/G-patch domain-containing protein
			AT4G29930	Basic helix-loop-helix (bHLH) DNA-binding superfamily protein
			AT5G53180	Polypyrimidine tract-binding protein 2, PBT2
			AT4G00440	GPI-anchored adhesin-like protein, putative DUF3741/TON1 Recruiting motif 15, TRM15
			AT4G35785	RNA-binding (RRM/RBD/RNP motifs) family protein
			AT5G65685	UDP-Glycosyltransferase superfamily protein
			AT1G06220	Maternal effect embryo arrest 5, MEE5
			AT1G77080	MADS affecting flowering 1, MAF1
			AT1G19080	GINS complex protein, TTN10
			AT1G30810	Jumonji domain-containing protein 18, JMJ18
			AT2G26980	CBL-interacting protein kinase 3, CIPK3
			AT1G16610	Arginine/Serine-rich, SR45
			AT5G24630	Brassinosteroid-insensitive 4, BIN4
			AT1G13450	Homeodomain-like superfamily protein, GT-1
			AT3G07650	CONSTANS-like 9, COL9
			AT4G38240	Complexglycanless, CGL1
			AT1G05230	Homeodomain GLABROUS 2, HDG2
			AT4G34265	Hypothetical protein
			AT5G10490	MSCS-like 2, MSL2
			AT3G26690	Nudix hydrolase homolog 13, NUDX13
Primary metabolic process	37	0.013	AT5G37370	PRP38 family protein, ATSRL1
			AT1G28610	GDSL-like Lipase/Acylhydrolase superfamily protein
			AT2G41540	GPDHC1
			AT3G17040	High-chlorophyll fluorescent 107, HCF107
			AT5G17300	REVEILLE 1, RVE1
			AT1G49340	Phosphatidylinositol 3- and 4-kinase family protein,ATPI4K ALPHA
			AT3G46600	GRAS family transcription factor
			AT4G20380	Lesion simulating disease 1, LSD1
			AT1G79500	Aldolase-type TIM barrel family protein,

	ATKDSA1
AT5G38290	Peptidyl-tRNA hydrolase family protein
AT1G43620	UDP-Glycosyltransferase superfamily protein, UGT80B1
AT1G30810	Jumonji domain-containing protein 18, JMJ18
AT1G54385	ARM repeat superfamily protein
AT5G37380	Chaperone DnaJ-domain superfamily protein
AT3G06340	DNAJ heat shock N-terminal domain-containing protein
AT4G12460	OSBP(oxysterol binding protein)-related protein 2B, ORP2B
AT3G07650	CONSTANS-like 9, COL9
AT3G27110	Peptidase family M48 family protein
AT5G24630	Brassinosteroid-insensitive 4, BIN4
AT1G13450	Homeodomain-like superfamily protein, GT-1
AT1G05230	Homeodomain GLABROUS 2, HDG2
AT1G01060	Late elongated hypocotyl, LHY1, LHY1
AT1G65120	Ubiquitin carboxyl-terminal hydrolase-related protein
AT4G23250	Cysteine-rich receptor-like protein kinase 17, CRK17
AT4G11830	phospholipase D gamma 2, PLDGAMMA2
AT5G47080	casein kinase II beta chain 1, CKB1
AT1G16610	Arginine/Serine-rich, SR45
AT5G56500	TCP-1/cpn60 chaperonin family protein
AT5G23210	Serine carboxypeptidase-like 34, SCPL34
AT4G29930	Basic helix-loop-helix (bHLH) DNA-binding superfamily protein
AT1G77080	MADS affecting flowering 1, MAF1
AT4G38240	Complexglycanless, CGL1
AT5G63370	Cyclin-dependent kinase G1, CDKG1
AT3G47340	Glutamine-dependent asparagine synthase 1, ASN1
AT3G02600	Lipid phosphate phosphatase 3, LPP3
AT1G19080	GINS complex protein, TTN10
AT3G01850	Aldolase-type TIM barrel family protein

2. ABA treatment

Function of genes	Number of genes	P value	Locus	Description
Response to abiotic stimulus	9	0.011	AT4G38240	Complexglycanless, CGL1
			AT5G37370	PRP38 family protein,ATSRL1
			AT5G65060	MADS affecting flowering 3, MAF3
			AT2G33380	Responsive to desiccation 20, RD20
			AT3G22420	With no lysine (K) kinase 2, WNK2
			AT5G48150	Phytochrome a signal transduction 1, PAT1
			AT1G77080	MADS affecting flowering 1, MAF1
			AT1G69530	Expansin A1, EXPA1
			AT3G47340	Glutamine-dependent asparagine synthase 1, ASN1
Response to light stimulus	5	0.017	AT3G22420	With no lysine (K) kinase 2, WNK2
			AT1G69530	Expansin A1, EXPA1
			AT3G47340	Glutamine-dependent asparagine synthase 1, ASN1
			AT5G48150	Phytochrome a signal transduction 1, PAT1
			AT1G77080	MADS affecting flowering 1, MAF1
Response to radiation	5	0.019	AT3G22420	With no lysine (K) kinase 2, WNK2
			AT1G69530	Expansin A1, EXPA1
			AT3G47340	Glutamine-dependent asparagine synthase 1, ASN1
			AT5G48150	Phytochrome a signal transduction 1, PAT1
			AT1G77080	MADS affecting flowering 1, MAF1
Post-embryonic development	6	0.0087	AT1G19080	GINS complex protein, TTN10
			AT1G77080	MADS affecting flowering 1, MAF1
			AT5G65060	MADS affecting flowering 3, MAF3
			AT5G45190	Cyclin family protein
			AT3G22420	With no lysine (K) kinase 2, WNK2
			AT5G48150	Phytochrome a signal transduction 1, PAT1
Nitrogen compound metabolic process	16	0.027	AT5G37370	PRP38 family protein,ATSRL1
			AT1G19080	GINS complex protein, TTN10
			AT1G61010	Cleavage and polyadenylation specificity faction 73-I, CPSF73
			AT3G18100	MYB domain protein 4R1, MYB4R1
			AT2G22570	Nicotinamidase 1, NIC1
			AT5G09330	NAC028
			AT5G65060	MADS affecting flowering 3, MAF3
			AT3G52120	Suppressor-of-White-APricot/surp domain-containing protein / D111/G-patch domain-containing protein, SWAP
			AT1G07350	RNA-binding (RRM/RBD/RNP motifs)

				family protein, SR45a
			AT1G05710	Basic helix-loop-helix (bHLH) DNA-binding superfamily protein
			AT5G23020	2-isopropylmalate synthase 2, IMS2
			AT1G77080	MADS affecting flowering 1, MAF1
			AT4G02120	CTP synthase family protein
			AT5G53460	NADH-dependent glutamate synthase 1, GLT1
			AT3G47340	Glutamine-dependent asparagine synthase 1, ASN1
			AT1G31360	RECQ helicase L2, RECQL2
Primary metabolic process	30	0.046	AT2G44065	Ribosomal protein L2 family
			AT5G37370	PRP38 family protein,ATSRL1
			AT1G43620	UDP-Glycosyltransferase superfamily protein, UGT80B1
			AT5G65060	MADS affecting flowering 3, MAF3
			AT2G33380	Responsive to desiccation 20, RD20
			AT3G22420	With no lysine (K) kinase 2, WNK2
			AT5G56500	TCP-1/cpn60 chaperonin family protein
			AT5G23210	Serine carboxypeptidase-like 34, SCPL34
			AT5G53460	NADH-dependent glutamate synthase 1, GLT1
			AT1G07350	RNA-binding (RRM/RBD/RNP motifs) family protein, SR45a
			AT5G66210	Calcium-dependent protein kinase 28, CPK28
			AT4G31170	Protein kinase superfamily protein
			AT1G05710	Basic helix-loop-helix (bHLH) DNA-binding superfamily protein
			AT1G77080	MADS affecting flowering 1, MAF1
			AT4G02120	CTP synthase family protein
			AT3G26085	CAAX amino terminal protease family protein
			AT4G38240	Complexglycanless, CGL1
			AT1G31360	RECQ helicase L2, RECQL2
			AT2G22570	Nicotinamidase 1, NIC1
			AT5G63370	Cyclin-dependent kinase G1, CDKG1
			AT3G52120	Suppressor-of-White-APricot/surp domain-containing protein / D111/G-patch domain-containing protein, SWAP
			AT5G23020	2-isopropylmalate synthase 2, IMS2
			AT3G47340	Glutamine-dependent asparagine synthase 1, ASN1
			AT3G02600	Lipid phosphate phosphatase 3, LPP3

			AT1G19080	GINS complex protein, TTN10
			AT3G18100	MYB domain protein 4R1, MYB4R1
			AT5G09330	NAC domain containing protein 82, NAC028
			AT1G61010	Cleavage and polyadenylation specificity faction 73-I, CPSF73
			AT3G01850	Aldolase-type TIM barrel family protein
			AT4G00560	NAD(P)-binding Rossmann-fold superfamily protein
Catalytic activity	36	0.0044	AT2G44065	Ribosomal protein L2 family
			AT1G28960	Nudix hydrolase homolog 15, NUDX15
			AT1G43620	UDP-Glycosyltransferase superfamily protein, UGT80B1
			AT2G35660	FAD/NAD(P)-binding oxidoreductase family protein, CTF2A
			AT3G63340	Kinase superfamily protein
			AT5G23210	Serine carboxypeptidase-like 34, SCPL34
			AT2G24270	Aldehyde dehydrogenase 11A3, ALDH11A3
			AT1G55120	Beta-fructofuranosidase 5, FRUCT5
			AT2G48100	Exonuclease family protein
			AT5G01670	NAD(P)-linked oxidoreductase superfamily protein
			AT4G31170	Protein kinase superfamily protein
			AT3G13440	S-adenosyl-L-methionine-dependent methyltransferases superfamily protein
			AT1G21350	Thioredoxin superfamily protein
			AT2G33380	Responsive to desiccation 20, RD20
			AT3G22420	With no lysine (K) kinase 2, WNK2
			AT5G53460	NADH-dependent glutamate synthase 1, GLT1
			AT5G66210	Calcium-dependent protein kinase 28, CPK28
			AT4G02120	CTP synthase family protein
			AT4G38240	Complexglycanless, CGL1
			AT1G31360	RECQ helicase L2, RECQL2
			AT2G22570	Nicotinamidase 1, NIC1
			AT5G63370	Cyclin-dependent kinase G1, CDKG1
			AT5G20250	Dark inducible10, DIN10
			AT5G23020	2-isopropylmalate synthase 2, IMS2
			AT3G47340	Glutamine-dependent asparagine synthase 1, ASN1
			AT3G02600	Lipid phosphate phosphatase 3, LPP3

	AT2G06025	Acyl-CoA N-acyltransferases (NAT) superfamily protein
	AT5G14610	DEAD box RNA helicase family protein
	AT5G45190	Cyclin family protein
	AT3G26690	Nudix hydrolase homolog 13, NUDX13
	AT4G23000	Calcineurin-like metallo-phosphoesterase superfamily protein
	AT5G65685	UDP-Glycosyltransferase superfamily protein
	AT5G22620	Phosphoglycerate/bisphosphoglycerate mutase family protein
	AT3G01850	Aldolase-type TIM barrel family protein
	AT3G63480	KINESIN 1, KIN1
	AT4G00560	NAD(P)-binding Rossman-fold superfamily protein
